# Distinct mechanisms define murine B cell lineage immunoglobulin heavy chain (IgH) repertoires

**DOI:** 10.7554/eLife.09083

**Published:** 2015-09-30

**Authors:** Yang Yang, Chunlin Wang, Qunying Yang, Aaron B Kantor, Hiutung Chu, Eliver EB Ghosn, Guang Qin, Sarkis K Mazmanian, Jian Han, Leonore A Herzenberg

**Affiliations:** 1 Genetics Department, Stanford University, Stanford, United States; 2 HudsonAlpha Institute for Biotechnology, Huntsville, United States; 3 Biology and Biological Engineering Department, California Institute of Technology, Pasadena, United States; National Institute of Immunology, India

**Keywords:** V(D)J, diversify, IgH, Mouse

## Abstract

Processes that define immunoglobulin repertoires are commonly presumed to be the same for all murine B cells. However, studies here that couple high-dimensional FACS sorting with large-scale quantitative IgH deep-sequencing demonstrate that B-1a IgH repertoire differs dramatically from the follicular and marginal zone B cells repertoires and is defined by distinct mechanisms. We track B-1a cells from their early appearance in neonatal spleen to their long-term residence in adult peritoneum and spleen. We show that de novo B-1a IgH rearrangement mainly occurs during the first few weeks of life, after which their repertoire continues to evolve profoundly, including convergent selection of certain V(D)J rearrangements encoding specific CDR3 peptides in all adults and progressive introduction of hypermutation and class-switching as animals age. This V(D)J selection and AID-mediated diversification operate comparably in germ-free and conventional mice, indicating these unique B-1a repertoire-defining mechanisms are driven by antigens that are not derived from microbiota.

**DOI:** http://dx.doi.org/10.7554/eLife.09083.001

## eLife digest

Our immune system protects us by recognizing and destroying invading viruses, bacteria and other microbes. B cells are immune cells that produce protective proteins called antibodies to stop infections. These cells are activated by ‘antigens’, which are fragments of molecules from the microbes or from our own cells. When an antigen binds to a B cell, the cell matures, multiplies and produces proteins called antibodies. These antibodies can bind to the antigen, which marks the microbe for attack and removal by other cells in the immune system.

Each antibody consists of two ‘heavy chain’ and two ‘light chain’ proteins. B cells are able to produce a large variety of different antibodies due to the rearrangement of the gene segments that encode the heavy and light chains. In mice, there are two kinds of B cells – known as B-1a and B-2 cells – that play different roles in immune responses. B-1a cells have long been known to produce the ‘natural’ antibodies that are present in the blood prior to an infection. On the other hand, B-2 cells produce antibodies that are specifically stimulated by an infection and are better adapted to fighting it. Previous studies have shown that both types of antibodies are required to allow animals to successfully fight the flu virus.

Here, Yang, Wang et al. used a technique called fluorescence-activated cell sorting (or FACS) and carried out extensive genomic sequencing to study how the B-1a and B-2 populations rearrange their genes to produce heavy chains. This approach made it possible to separate the different types of B cells and then sequence the gene for the heavy chain within the individual cells. The experiments show that the “repertoire” of heavy chains in the antibodies of the B-1a cells is much less random and more repetitive than that of B-2 populations.

Furthermore, Yang, Wang et al. show that B-1a cells produce and maintain their repertoire of heavy chains in a different way to other B-2 populations. B-1a cells develop earlier and the major genetic rearrangements in the gene that encodes the heavy chain occur within the first few weeks of life. Although the gene rearrangements have mostly stopped by adulthood, the B-1a antibody repertoire continues to evolve profoundly as the B-1a cells divide over the life of the animal. On the other hand, the gene rearrangements that make the heavy chains in the B-2 cells continue throughout the life of the animal to produce the wider repertoire of antibodies found in these cells. In addition, the processes that continue to change the antibody reperotire in the B-1a cells during adulthood do not occur in the B-2 populations.

Importantly, the these reperotire-changing processes in B-1a cells also occur in mice that have been raised in germ-free conditions, which demonstrates that – unlike other B cells – the repertoire of heavy chains in B-1a cells is not influenced by antigens from microbes. Instead, it is mainly driven by antigens that are expressed by normal cells in the body. These findings open the way to future work aimed at understanding how B-1a cells help to protect us against infection, and their role in autoimmune diseases, where immune cells attack the body’s own healthy cells.

**DOI:** http://dx.doi.org/10.7554/eLife.09083.002

## Introduction

Follicular B (FOB), marginal zone B (MZB) and B-1a cells are the major mature B cell populations in the mouse. Although these B cell subsets all produce functionally important antibodies, they differ profoundly in function and developmental origin (Kantor and Herzenberg, 1993; Hardy and Hayakawa, 2001; Baumgarth, 2011). Previous studies have shown that B-1a cells are efficiently generated during fetal and neonatal life, and are maintained by self-replenishment in adult animals(Hayakawa et al., 1985; Montecino-Rodriguez et al., 2006; Kantor et al., 1992). In contrast, both FOB and MZB populations emerge later and are replenished throughout life by *de novo* development from bone marrow (BM) hematopoietic stem cells (HSC). Our recent studies show that BM HSC reconstitute FOB and MZB, but fail to reconstitute B-1a cells (Ghosn et al., 2012), which are derived from distinct progenitors at embryonic day 9 yolk sac (Yoshimoto et al., 2011).

For each B cell subset, their antibody responses are enabled by the basic processes that generate the immunoglobulin (Ig) structure. Multiple mechanisms contribute to creating the primary Ig heavy (IgH) and light chain (IgL) diversity. For IgH, these include combinatorial assortment of individual variable (V), diversity (D) and joining (J) gene segments, nucleotide(s) trimming in the D-J and V-DJ joining site, and, template-dependent (P-addition) and independent (N-addition) nucleotide(s) insertion at the joined junctions (Yancopoulos and Alt, 1986; Kirkham and Schroeder, 1994). The V(D)J joining processes define the third IgH complementarity-determining region (CDR3), which often lies at the center of antigen binding site and plays a crucial role in defining antibody specificity and affinity (Xu and Davis, 2000).

After encountering antigen, “‘naïve”’ B cells are activated and can further diversify their primary antibody repertoire by activation-induced cytidine deaminase (AID)–mediated somatic hypermutation (SHM), which introduces single or multiple mutations into the IgV regions (Muramatsu et al., 2000; Wagner and Neuberger, 1996). SHM commonly occurs in germinal centers (GC) (Victora and Nussenzweig, 2012), where memory B cells expressing high affinity antibodies are selected (Rajewsky, 1996; Gitlin et al., 2014). Since the antigen-driven SHM-mediated secondary Ig diversification is viewed as a crucial adaptation to the environmental needs, the IgH repertoire(s) expressed by FOB, MZB and B-1a cells from non-immunized animals are thought to be free of SHM. Our studies here, however, introduce a previously unrecognized SHM mechanism that increasingly diversifies the B-1a pre-immune IgH repertoire as animals age. Importantly, the SHM operates equally in the presence or absence of microbiota influence.

The B-1a antibody repertoire is commonly thought to be ‘restricted’ with expressing germline genes, largely because the hybridomas generated from fetal and neonatal B cells, which are mainly B-1a, have few N-insertions (Carlsson and Holmberg, 1990) and preferentially express the proximal 7183, Q52 V_H_ family genes (Perlmutter et al., 1985). The N diversity deficit is ascribed to the absence of expression of terminal deoxynucleotidyl transferase (*Tdt*), which adds the N nucleotides to the CDR3 junction (Gilfillan et al., 1993), during fetal life (Feeney, 1990). These early studies left the impression that the proximal V_H_ gene usage predominates and that there is little N-addition in the B-1a IgH repertoire.

Later studies by the Rajewsky group, however, showed that although neonatal (4 day) splenic B-1a cells contain very few N-insertions, N addition is readily detected in substantial numbers of peritoneal B-1a cells from adult animals (Gu et al., 1990), indicating that B-1a cells are continuously generated after *Tdt* is expressed. Holmberg lab similarly found the low N-region diversity in the adult peritoneal B-1a repertoire (Tornberg and Holmberg, 1995). Our early studies confirm and extend these findings by showing that roughly two thirds of the IgH sequences from individually sorted peritoneal B-1a cells have N additions (Kantor et al. 1997). Furthermore, recent studies have shown that B-1a progenitors from both fetal liver and adult BM sources generate peritoneal B-1a cells with substantial N-addition (Holodick et al., 2014). Collectively, these findings demonstrate that the peritoneal B-1a IgH repertoire diversity is greater than previously thought.

However, these studies mainly characterized the repertories of B cells in the peritoneal cavity (PerC) and leave the questions open as to whether and how the repertoire changes throughout ontogeny in B cells at various sites of development and function. Studies here address these issues. We show that the B-1a IgH repertoire differs drastically from the repertories expressed by splenic FOB, MZB and peritoneal B-2 cells. In addition, we track the development of B-1a cells from their early appearance in neonatal spleen to their long-term residence in adult peritoneum and spleen, and elucidate the previous unrecognized somatic mechanisms that select and diversify the B-1a IgH repertoire over time. Most importantly, the potent mechanisms that uniquely act in B-1a (not in FOB and MZB cells) operate comparably in germ-free (GF) and conventional mice reared under specific pathogen free (SPF) condition, indicating that these repertoire-defining mechanisms are not driven by microbiota-derived antigens.

The dearth of these advanced understandings in the previous studies is largely due to technical difficulties that limited both their scope and depth. Studies analyzing Ig sequences from immortalized cell lines (e.g., hybridomas) or LPS-stimulated B cells had obvious sampling biases. In addition, earlier studies mainly focused on particular V_H_ families (e.g., J558, 7183), even though the mouse IgH locus contains over 100 functional V_H_ genes (Kirkham and Schroeder, 1994). The introduction of single cell analyses enabled higher precision and lower bias than the bulk measurements. However, they were constrained profoundly by sequencing costs and technical challenges. Indeed, our previous single cell analysis reported only 184 IgH sequences derived from 85% recovered sorted single cells representative of three types of peritoneal B subsets (Kantor et al., 1997). Thus, while the data yielded key insights, hundreds or thousands of single cells would need to be analyzed to obtain a more comprehensive view for a single B subset repertoire. Finally, difficulties in defining and cleanly sorting rare B subsets (e.g., splenic B-1a) further compromise the attempt to develop a thorough view of repertoire(s) expressed by various B cell subsets at the different anatomic location and ontogenic stage.

To overcome these obstacles, we have coupled high-dimensional (Hi-D) FACS sorting with unique IgH multiplex PCR technologies, which allow inclusive amplification of IgH transcripts for each sorted B subset and ultimate sequencing of these sequences. Using barcoded sample multiplexing, we have performed a large-scale quantitative and comparative study of the ‘pre-immune’ IgH repertoires expressed by various functionally and developmentally distinct mature B subsets (splenic FOB, MZB and B-1a; peritoneal B-2 and B-1a) from non-immune C57BL/6J mice. In addition, since microbiota are often thought to influence the Ig repertoire, we have compared the B-1a IgH repertoires in GF or conventional mice.

## Results

### The B-1a pre-immune IgH repertoire is far more restricted and repetitive than the repertoire expressed by FOB and MZB subsets

**Figure 1.**
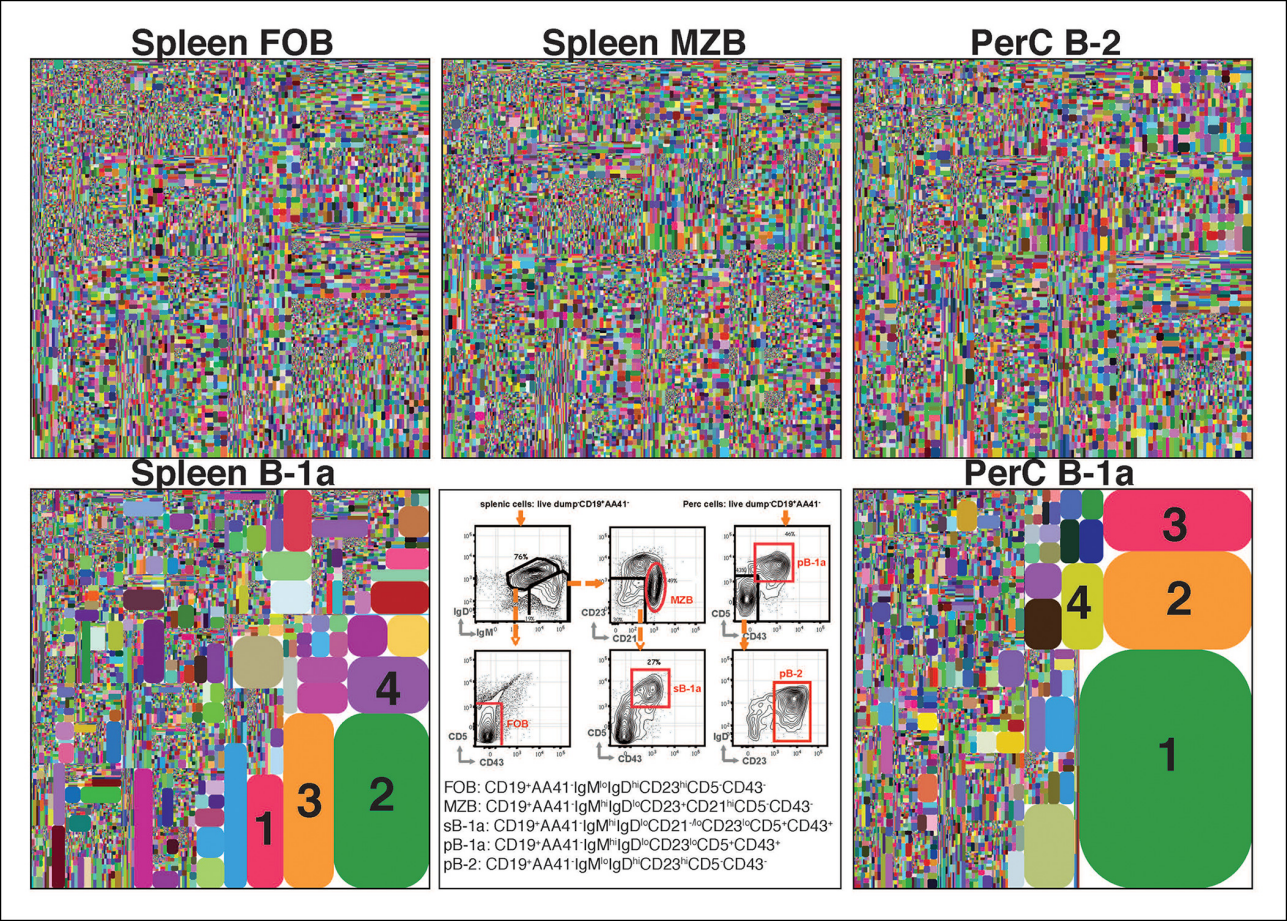
The B-1a IgH CDR3 sequences are much less diverse and recur more frequently than the CDR3 sequences expressed by FOB and MZB B subsets. IgH CDR3 tree-map plots illustrating the IgH CDR3 nucleotide sequences expressed by indicated B cell subsets sorted from one 2-month old C57Bl/6 mouse. Each rectangle in a given tree-map represents a unique CDR3 nucleotide sequence and the size of each rectangle denotes the relative frequency of an individual sequence. The colors for the individual CDR3 sequences in each tree-map plot are chosen randomly thus do not match between plots. The numbers shown in the CDR3 tree-map plots highlight the highly reoccurring CDR3 sequences including PtC-binding CDR3 sequences. 1, ARFYYYGSSYAMDY, V1-55D1-1J4; 2, MRYGNYWYFDV, V11-2D2-8J1; 3, MRYSNYWYFDV, V11-2D2-6J1; 4, MRYGSSYWYFDV, V11-2D1-1J1. *Lower middle panel*: FACS plots showing the gating strategy used to sort the phenotypically defined each B cell subset from spleen (s) or peritoneal cavity (p). Note: peritoneal B-1a cells are well known to express CD11b, a marker expressed on many myeloid cells including macrophage and neutrophils. The level of CD11b expressed on peritoneal B-1a cells, however, is roughly 100 fold lower than the level of CD11b expressed on the myeloid cells. This drastic difference is sufficient to separate the CD11b^+^ B-1a cells from the myeloid cells if monoclonal anti-CD11b reagent is included in the dump channel (Figure 1—figure supplement 3). **DOI:** http://dx.doi.org/10.7554/eLife.09083.003

We sorted splenic and peritoneal B-1a (dump^-^ CD19^+^ CD93^-^IgM^hi^ IgD^lo/-^ CD21^-/lo^ CD23^-^ CD43^+^ CD5^+^); splenic FOB and peritoneal B-2 (dump^-^ CD19^+^ CD93^-^ IgM^lo^ IgD^hi^ CD23^+^ CD43^-^ CD5^-^); and splenic MZB (dump^-^ CD19^+^ CD93^-^ IgM^hi^ IgD^lo/-^ CD21^hi^ CD23^lo/-^ CD43^-^ CD5^-^) from non-immune C57BL/6 mice (Figure 1). We generated and amplified IgH cDNA libraries from each subset. We then pooled the libraries, which are distinguishable by barcode, and sequenced them (Illumina MiSeq). In all, we sequenced 60 separately prepared libraries, each derived from 1-2 x10^4^ B cells of a given subset sorted from mice at the same or different ages (from 2 days to 6 months, > 30 mice) (Table 1). Overall 18 million total clean nucleotide sequences (CNT) and about half million unique clean nucleotide sequences (CNU) were analyzed in the study (Table 1).

**Figure 1—figure supplement 1.**
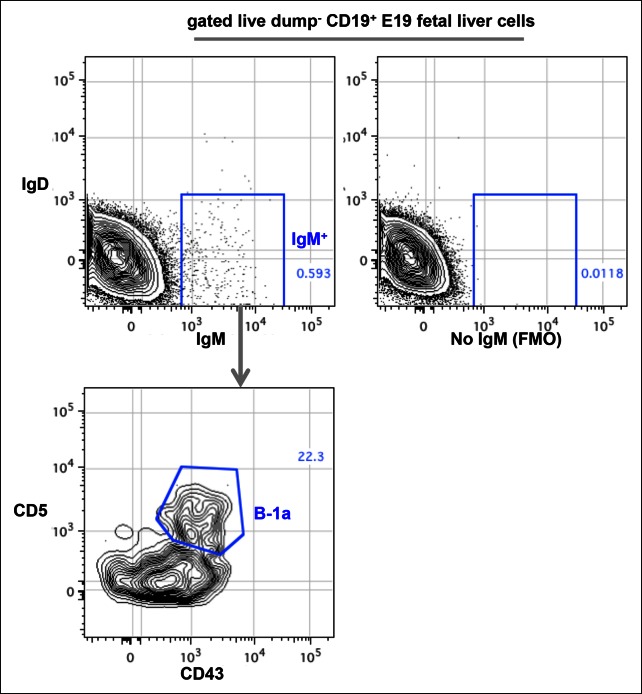
FACS plots showing CD43^+^ CD5^+^ IgM^+^ B-1a cells in E19 fetal liver. Live dump^-^ (CD11b^-^ CD11c^-^ Gr-1^-^ F4/80^-^ CD3^-^ TCRαβ^-^) CD45^+^ CD19^+^ cells from E19 fetal liver of C57Bl/6 mouse were gated to show IgM and IgD expression. The boundary for IgM expression was determined from fluorescence-minus-one (FMO) control in which fluorescently labeled anti-mouse IgM antibodies are omitted from the staining sets (right plot). IgM^+^ IgD^-^ cells were further gated to reveal CD43^+^ CD5^+^ B-1a cells. **DOI:** http://dx.doi.org/10.7554/eLife.09083.004

**Figure 1—figure supplement 2.**
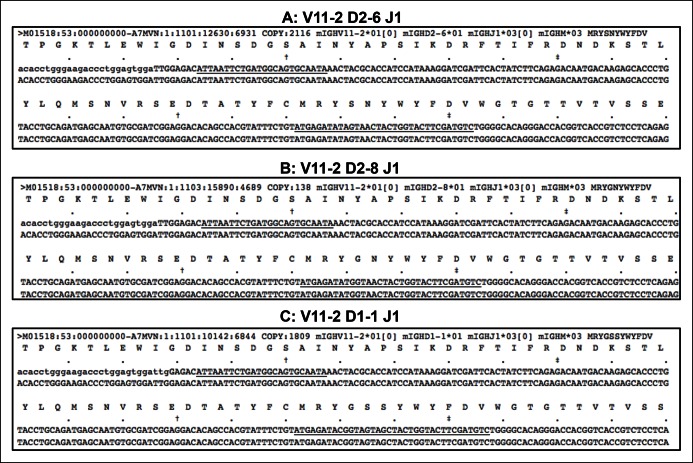
Recurrent V_H_11-encoded PtC-binding V(D)J sequences. (**A-C**) lists three V_H_11-encoded PtC-binding V(D)J sequences. In each plot, the first line of nucleotides is the obtained sequence read while the second line refers the germline reference sequence. The underlined nucleotides are CDR2 and CDR3. **DOI:** http://dx.doi.org/10.7554/eLife.09083.005

**Figure 1—figure supplement 3.**
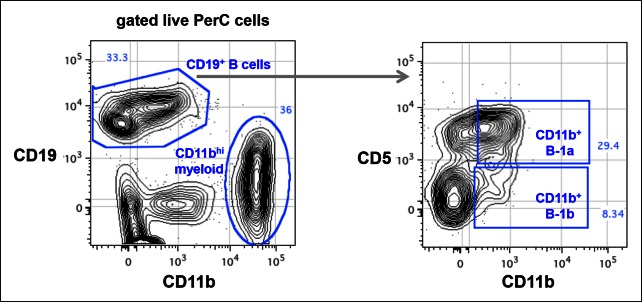
CD11b expression on peritoneal B-1a (CD5^+^) and B-1b (CD5^-^) is roughly 100-fold lower than the CD11b expression on myeloid cells. Live cells from C57Bl/6 peritoneal cavity were gated to show CD19 and CD11b expression. The CD19 ^+^ B cells and CD11b^hi^ myeloid cells were shown. The CD19^+^ B cells were gated to reveal CD5 and CD11b expression. CD11b^+^ B-1a and CD11b ^+^ B-1b cells were gated based on FMO control staining where anti-CD11b antibody was omitted in the staining. **DOI:** http://dx.doi.org/10.7554/eLife.09083.006

**Table 1.** Summary of the sequences for 60 separately sorted B cell populations analyzed in this study. **DOI:** http://dx.doi.org/10.7554/eLife.09083.007

Sample	Id	Subset	Strain	Age	Condition	Mice	RNT*	RNU*	RPU*	CNT*	CNU*	CPU*
1	7631	FOB	WT	2M	SPF	single	1006030	151210	65871	903400	21240	20470
2	13966	FOB	WT	3.5M	SPF	single	150812	31003	24801	130652	14911	14678
3	8706	FOB	WT	4M	SPF	single	180365	53577	27817	159710	16901	16568
4	8702	FOB	WT	5M	SPF	single	156681	54195	27728	136101	16951	16649
5	13967	FOB	AID KO	5M	SPF	single	35967	14623	13203	27726	7187	7133
6	11161	MZB	WT	1M	SPF	single	33548	19628	12744	25674	6584	6471
7	10658	MZB	WT	2M	SPF	single	71458	26978	18278	61258	11512	11170
8	7630	MZB	WT	2M	SPF	single	1032381	139832	62520	932353	20780	19792
9	8701	MZB	WT	4M	SPF	single	214238	55075	26458	191065	15461	15021
10	8700	MZB	WT	5M	SPF	single	118863	42310	22794	102894	14517	14180
11	13338	MZB	WT	4M	GF	single	162754	39930	23611	141646	12939	12605
12	13343	MZB	WT	4M	GF	single	595780	85497	45820	536072	19266	18480
13	11163	pB-1a	WT	1M	SPF	pool of 3 mice	45882	11290	5596	41368	3237	3007
14	10660	pB-1a	WT	2M	SPF	single	222324	17311	8630	207749	3891	3649
15	13018	pB-1a	WT	2M	SPF	single	808879	36031	14817	753868	4769	4374
16	7628	pB-1a	WT	2M	SPF	single	1784677	59458	22105	1706235	6601	5848
17	11160	pB-1a	WT	2W	SPF	pool of 8 mice	65317	14700	7025	58034	4240	3704
18	10655	pB-1a	WT	3W	SPF	pool of 5 mice	62875	12162	6622	57558	4180	3694
19	8705	pB-1a	WT	4M	SPF	single	310077	28441	11886	287695	5063	4707
20	9870	pB-1a	WT	4M	SPF	single	229100	26299	10469	211514	4745	4480
21	11165	pB-1a	WT	5M	SPF	single	105410	19528	8926	95994	4435	4162
22	8707	pB-1a	WT	5M	SPF	single	320252	29786	12423	296946	4722	4384
23	9861	pB-1a	WT	6M	SPF	single	26613	5683	3235	23542	1521	1461
24	8704	pB-1a	AID KO	4M	SPF	single	264340	33745	14519	245941	6648	6294
25	10657	pB-2	WT	2M	SPF	single	53953	23059	16883	44986	10084	9923
26	7629	pB-2	WT	2M	SPF	single	1315663	123472	47337	1238225	16925	16065
27	13969	pB-2	WT	3.5M	SPF	single	186817	24304	17689	170768	9089	8925
28	9862	pB-2	WT	4M	SPF	single	22591	13377	8737	17343	4382	4357
29	13973	pB-2	AID KO	5M	SPF	single	617893	62319	41165	566826	17536	16965
30	13000	sB-1a	WT	2d	SPF	pool of 8 mice	29439	9542	4925	25369	3148	2758
31	10651	sB-1a	WT	5d	SPF	single	123360	22472	10838	113161	7453	5976
32	10659	sB-1a	WT	5d	SPF	single	210055	28140	12411	192662	7307	5812
33	9866	sB-1a	WT	5d	SPF	single	52986	15600	6864	46580	4595	3837
34	10652	sB-1a	WT	6d	SPF	single	172875	26437	12545	159304	7683	6365
35	9865	sB-1a	WT	7d	SPF	single	71309	18446	8775	64241	5482	4941
36	9868	sB-1a	WT	7d	SPF	single	201813	35069	14473	186227	7847	6843
37	10656	sB-1a	WT	2M	SPF	single	369732	39603	19759	342914	9489	9048
38	13004	sB-1a	WT	2M	SPF	single	185948	27952	13875	168522	7313	7022
39	7632	sB-1a	WT	2M	SPF	single	1825218	102797	43190	1719246	12428	11144
40	11168	sB-1a	WT	2W	SPF	single	536603	70201	28829	496671	11948	10913
41	13005	sB-1a	WT	2W	SPF	single	98017	28331	15001	85489	8820	8207
42	10654	sB-1a	WT	3W	SPF	single	146560	33814	19697	131091	11995	11451
43	13970	sB-1a	WT	3.5M	SPF	single	170925	13809	9289	160480	4513	4273
44	13335	sB-1a	WT	4M	SPF	single	22175	4822	3449	18683	1131	1090
45	13342	sB-1a	WT	4M	SPF	single	283072	23668	12947	262744	5357	5032
46	8699	sB-1a	WT	4M	SPF	single	142838	19151	9938	130915	4370	4086
47	9863	sB-1a	WT	4M	SPF	single	73676	16599	8713	65571	4233	4092
48	11167	sB-1a	WT	5M	SPF	single	501367	38912	17336	463863	7573	7163
49	8708	sB-1a	WT	5M	SPF	single	577114	52723	22272	531508	9146	8441
50	9867	sB-1a	WT	6M	SPF	single	113492	20612	10625	101791	4563	4343
51	13965	sB-1a	AID KO	4M	SPF	single	177782	16419	12281	164189	6539	6293
52	13971	sB-1a	AID KO	4M	SPF	single	517141	34159	22031	482543	8966	8395
53	13968	sB-1a	AID KO	5M	SPF	single	427671	30839	20510	396974	9162	8545
54	13972	sB-1a	AID KO	5M	SPF	single	706116	36217	23255	660874	9294	8744
55	13001	sB-1a	WT	4M	GF	single	43507	8734	4855	38947	2318	2249
56	13002	sB-1a	WT	4M	GF	single	47203	8683	4820	42279	2053	1965
57	13003	sB-1a	WT	4M	GF	single	213347	22246	11068	197769	4705	4449
58	13017	sB-1a	WT	4M	GF	single	532250	40497	17375	501908	7019	6398
59	13337	sB-1a	WT	4M	GF	single	28559	6322	4417	24047	1544	1486
60	13341	sB-1a	WT	4M	GF	single	388208	28942	14837	360727	5674	5144
Id is a unique identifier for the sequence run												
RNT*, total raw nucleotide sequences												
RNU*, unique raw nucleotide sequences												
RPU*, unique raw peptide sequences												
CNT*, total clean nucleotide sequences												
CNU*, unique clean nucleotide sequences												
CPU*, unique clean peptide sequences												

Sequence statistics		RNT*		RNU*		RPU*		CNT*		CNU*		CPU*
Total		1.9E + 07		2.1E + 06		1.1E + 06		1.8E + 07		4.9E + 05		4.7E + 05
Mean		319865		35610		17848		295174		8233		7762
% CV		122		86		74		125		61		63

We also attempted to analyze the B-1a repertoire in fetal liver but found that there were too few B-1a cells to reliably sequence with our method. In essence, FACS analysis of embryonic day 19 (E19) fetal liver cells shows that IgM^+^ B cells represent only 0.6% of CD19^+^ total B cells and that only around 20% of these IgM^+^ B cells express the B-1a CD43^+^ CD5^+^ phenotype (Figure 1—figure supplement 1). The frequencies of IgM^+^ B cell in E18 fetal liver are even lower (0.2% of CD19^+^ B cells). These numbers are too low for us to recover enough material for sequencing from a feasible number of embryos.

The IgH CDR3 tree maps for each B cell subset show that splenic FOB and peritoneal B-2 cells express highly diversified IgH CDR3 nucleotide sequences, as do MZB cells (Figure 1). In contrast, CDR3 nucleotide sequences expressed by B-1a cells from either spleen or PerC are far less diverse and recur much more frequently (Figure 1). The recurrent CDR3 sequences include the well-studied V_H_11-encoded sequences specific for phosphatidylcholine (PtC) (Figure 1—figure supplement 2) and known to occur frequently in B-1a cells (Mercolino et al., 1988; Hardy et al., 1989; Seidl et al., 1997).

**Figure 2.**
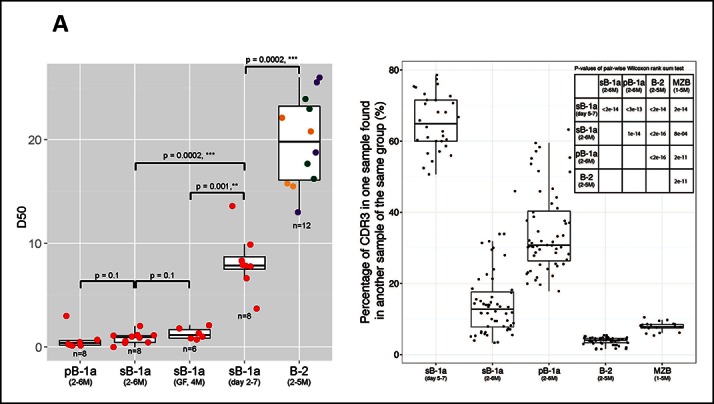
The B-1a pre-immune IgH repertoire is far more restricted than the pre-immune IgH repertoires expressed by splenic FOB, MZB and peritoneal B-2 cells. (**A**) D50 metric analysis quantifying the IgH CDR3 diversity for B cell subsets from mice at the indicated age. Low D50 values are associated with less diversity. Each dot represents the data for a B cell sample from an individual mouse except for the 2 day splenic B-1a data, which are derived from sorted cells pooled from 8 mice. B-1a samples are labeled with red; B-2 samples include FOB (green, n = 4), pB-2 (purple, n = 4) and MZB (yellow, n = 4). The data for germ-free (GF) animals is discussed at the end of the Result section. (**B**) CDR3 peptide pair-wise sharing analysis of IgH repertoire similarity among multiple samples for each B cell group (n = 5-9). Each dot represents the percentage of common CDR3 peptides in one sample that are also found in another sample within a given group. For example, to compute the similarity between sample A and B, the percentage of CDR3 peptides in sample A that are also found in sample B ($p_{A\;\to \;B\;}$), together with the percentage of CDR3s in sample B that are also in sample A ($p_{B\to A}$) are used as an indicator. For comparison of 6 splenic B-1a samples in 5-7 day group, there are 30 comparisons. *Right upper:* p values showing the statistical significance between two groups. Box plots represent the 10^th^, 25^th^, 50^th^, 75^th^ and 90^th^ percentiles here and in other figures. **DOI:** http://dx.doi.org/10.7554/eLife.09083.008

D50 metric analysis quantifying the IgH CDR3 nucleotide sequence diversity shows that the IgH CDR3 nucleotide sequences expressed by the FOB and MZB subsets are significantly more diverse than those expressed by splenic and peritoneal B-1a cells (p = 0.0002, Mann-Whitney-Wilcoxon Test) (Figure 2A). Consistent with this finding, IgH CDR3 peptide pairwise sharing analysis, which measures the similarity of IgH CDR3 peptide expression for each B cell subset sorted from different mice, shows that the same CDR3 peptide sequences frequently appear in both splenic and peritoneal B-1a cells from different mice whereas the common CDR3 peptides are rare in FOB and MZB subsets (Figure 2B). Taken together, these data demonstrate that the B-1a pre-immune IgH repertoire is far more restricted and repetitive than IgH repertoires expressed by FOB and MZB subsets.

### V_H_ gene usage differs among the B-1a, FOB and MZB pre-immune IgH repertoires

We quantified the frequency of IgH sequences expressing individual V_H_ gene for each sorted B cell sample and then compared the V_H_ gene usage between two B cell subsets. B-1a cells are well-known to undergo self-replenishing in adult (Kantor et al., 1995). To minimize the impact of clonal expansion on the V_H_ gene usage profile, we collected normalized data, in which we scored each distinct IgH CDR3 nucleotide sequence expressing a given V_H_ gene as one, no matter how many times this sequence was detected.

**Figure 3.**
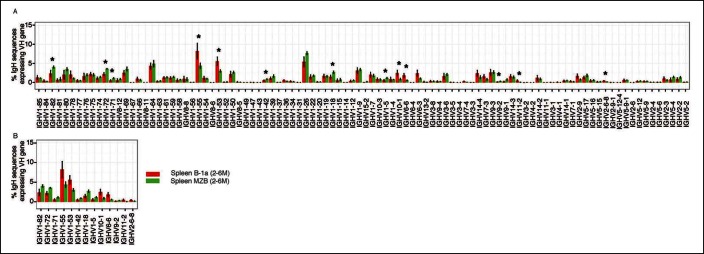
Comparison of V_H_ gene usage by splenic B-1a vs MZB B cells. (**A**) V_H_ gene usage profile shown as the percentage of IgH sequences expressing the listed individual V_H_ genes for individual B cell samples. The profiles are shown for adult splenic B-1a samples (n = 9, red) and for MZB samples (n = 5, green). V_H_ genes (from left to right) are ordered in 5’- to 3’-direction bases on chromosome location; the IMGT V_H_ gene nomenclature is used (Lefranc, 2003). (**B**) V_H_ genes showing the statistically significant differences (Welch’s t-test p<0.05) between two groups are listed and also highlighted with asterisks in the plot. To minimize the impact of the clonal expansion on the V_H_ gene usage profile, data are presented as the normalized distribution that counts each distinct CDR3 nucleotide sequence expressing a given V_H_ gene as one, no matter how many times the sequence was detected. Note: V_H_12-3 encoded IgH sequences are not detected in this study due to the technical limitations that exclude the V_H_12-3 primer from the set of primers designed about three years ago and used for studies presented here. We have since corrected this problem so that V_H_12-3 primer is now part of our new set of primers. Comparison of sequence data obtained with old vs. the new set of primers shows that, aside from now detecting V_H_12-3 sequences with the new set of primers, the sequences obtained with both primer sets are highly similar (Figure 3—figure supplement 2). **DOI:** http://dx.doi.org/10.7554/eLife.09083.009

Our approach enables detection of Ig transcripts expressing about 100 different V_H_ genes that belong to 14 V_H_ families (Figure 3). B-1a cells express all of these detected V_H_ genes (Figure 3A), contrasting with earlier impressions, based largely on hybridomas sequences from fetal and neonatal mice (Malynn et al., 1990), that V_H_ usage in the B-1a repertoire is very restricted. However, despite the broad V_H_ usage, certain V_H_ genes, notably V10-1 (DNA4), V6-6 (J606), V11-2 (V_H_11) and V2-6-8 (Q52), are expressed at a significantly higher frequency in splenic B-1a than MZB cells (p<0.05, Welch's t-test, Figure 3B).

**Figure 3—figure supplement 1.**
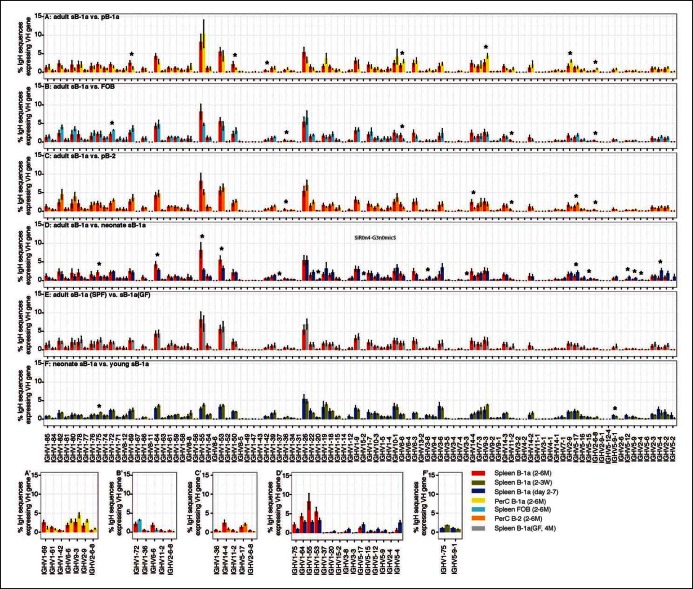
V_H_ gene usage profile pair-wise comparison of B cell groups. The colors shown at the bottom right distinguish the B cell groups (n = 4-9). V_H_ genes showing the statistically significant differences (Welch’s t-test p<0.05) between two groups are listed on the bottom (**A' to F'**) and also highlighted with asterisks in each plot. The data for germ-free (GF) animals is discussed at the end of the Result section. **DOI:** http://dx.doi.org/10.7554/eLife.09083.010

**Figure 3—figure supplement 2.**
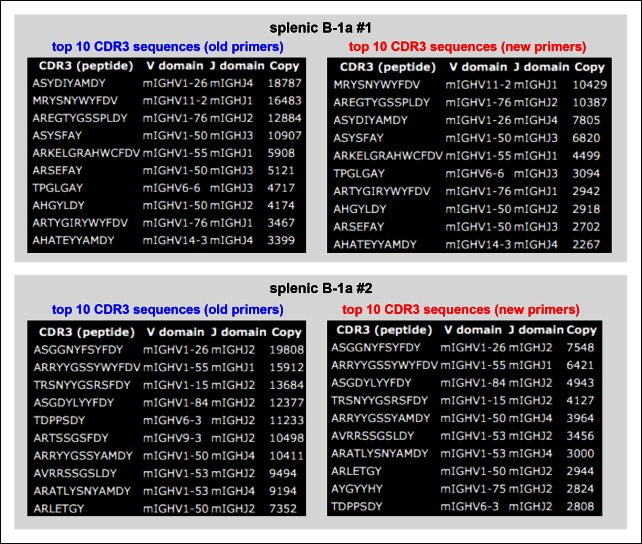
Almost identical top 10 highly recurring CDR3 sequences are detected for splenic B-1a IgH libraries obtained either with the old or new primer set. We sorted two splenic B-1a populations individually from two 4 month old C57BL/6J mice. We extracted RNA from each population and divided each RNA into two parts. For one part, we prepared an amplified library using the old primer set; and for the other, we prepared an amplified library using the new primer set. We then sequenced these amplified IgH libraries. Analysis of the resultant sequences showed that the sequences obtained from the IgH libraries are highly similar, regardless of the primers used (old or new). In essence, the top 10 highly recurring CDR3 sequences (both peptide and V(D)J recombination) are almost identical and show similar representation order between each pair of libraries. As expected, we detected V_H_12-3 encoded sequences from the splenic B-1a IgH libraries prepared with the new primer set, and these V_H_12-3 encoded sequences included several published PtC-binding V_H_12-3 encode sequences, i.e., AGDYDGYWYFDV (V_H_12-3D2-4J1), AGDRDGYWYFDV (V_H_12-3D3-2J1), AGDRYGYWYFDV (V_H_12-3 D2-9 J1). **DOI:** http://dx.doi.org/10.7554/eLife.09083.011

Similar to MZB cells, splenic FOB and peritoneal B-2 cells show lower frequency in expressing these B-1a favored V_H_ genes, i.e., V6-6 (J606), V11-2 (V_H_11) and V2-6-8 (Q52) (Figure 3—figure supplement 1B–C). Conversely, these B subsets tend to preferentially use the largest V_H_ family, V1 (J558), located distal to D_H_ and J_H_ gene segments (Yancopoulos and Alt, 1986). MZB cells, in particular, have a higher tendency to express certain V1 (J558) family genes including V1-82, V1-72, V1-71, V1-42, V1-18 and V1-5 (Figure 3B).

The V_H_ usage in the peritoneal B-1a cells is further biased toward V6-6 (J606), V9-3 (Vgam3.8), V2-9 (Q52) and V2-6-8 (Q52) genes, which are already favored in the splenic B-1a cells (Figure 3—figure supplement 1A). This finding indicates that the splenic and peritoneal B-1a populations are not in equilibrium and the latter is further enriched for cells expressing certain V_H_ genes.

### The B-1a IgH repertoire integrates rearrangements from de novo B-1a development that occur mainly during the first few weeks of life

Unlike FOB and MZB subsets, *de novo* B-1a development initiates prior to birth and decreases to a minimum in adult animals (Lalor et al., 1989; Barber et al., 2011). B-1a cells persist thereafter as a self-replenishing population (Kantor et al., 1995). To minimize the impact of self-replenishment on the N-addition distribution profile, and hence to weight the repertoire for de novo generated IgH sequences for B-1a cells, we collected normalized data that counts each distinct IgH sequence containing indicated N nucleotide insertions as a single sequence, regardless how many times this sequence was detected.

**Figure 4.**
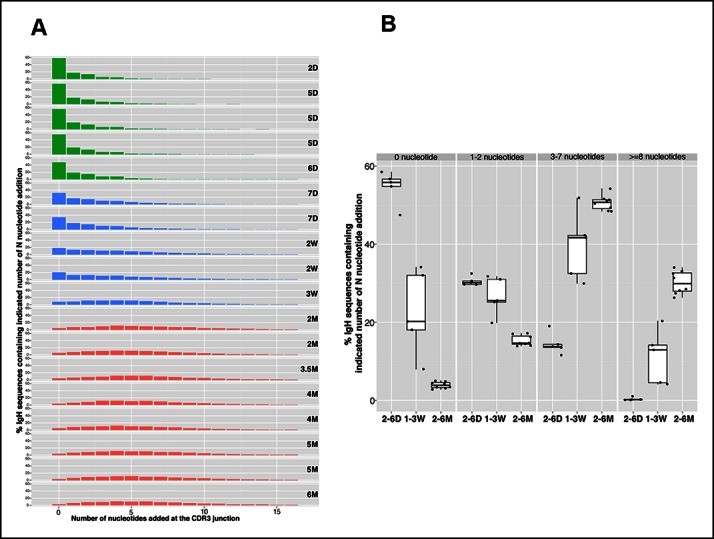
N nucleotide insertion distribution patterns for the B-1a pre-immune IgH repertoires during ontogeny. (**A**) Percentage of IgH sequences containing the indicated number of N nucleotide insertions at the IgH CDR3 junctions (V-DJ + D-J) is shown for each spleen B-1a sample from mice at indicated ages (shown at the right). To minimize the impact of self-renewal on the N-addition profile, normalized data are presented. Thus, each distinct IgH sequence containing indicated N nucleotide insertions is counted as one regardless how many times this sequence was detected. Note that the N insertion pattern changes as animals age. Colors distinguish three age-related patterns: green, D2 to D6; blue, D7 to 3W; red, 2M to 6M. (**B**) Percentages of IgH sequences containing the indicated N-nucleotide insertions (shown at the top) for splenic B-1a samples at the indicated ages are shown. Each dot represents data from an individual mouse, except for day 2 sample, n = 5-7. **DOI:** http://dx.doi.org/10.7554/eLife.09083.012

Consistent with *Tdt* expression, which is absent during the fetal life and initiates shortly after birth (Feeney, 1990; Bogue et al., 1992), N nucleotide insertion analysis of the splenic B-1a IgH repertoires demonstrate that roughly 60% of IgH sequences expressed by splenic B-1a cells from 2-–6 day mice do not contain N insertions at IgH CDR3 junction (D-J and V-DJ); about 30% contain 1–2 insertions; and, <15% contain 3–4 N-nucleotide insertions (Figure 4A,B). After 6 days, however, the frequency of sequences containing >3 N-additions progressively increases until the animals are weaned (roughly 3 weeks) (Figure 4A,B). After weaning, the N-addition pattern stabilizes, i.e., about 50% IgH sequences contain 3–7 N nucleotide insertions and about 30% have more than 8 N nucleotide insertions at IgH CDR3 junctions, and remains stable at this level for at least 5 months (Figure 4A,B).

In essence, splenic B-1a cells from 2-6 day mice largely originate from fetal and early neonatal wave(s) of B-1a development when *Tdt* is poorly expressed. As newborns progress to maturity, B-1a cells, which are originated in the earlier wave(s), are ‘diluted’ by B-1a cells that emerge during later development. The high frequency of N nucleotide additions in the adult splenic B-1a IgH repertoire indicates that a higher proportion of B-1a cells are actually generated postnatally after *Tdt* is expressed.

Cohering with the increased N diversity in the adulthood, CDR3 peptide pairwise sharing analysis shows that the expression of common IgH CDR3 peptides is significantly more frequent in neonatal splenic B-1a cells than in adult splenic B-1a cells (p<2e-16, Mann-Whitney-Wilcoxon Test, Figure 2B). V_H_ usage also shifts as animals mature. Splenic B-1a cells from neonatal mice (2-–7 days) preferentially express the V3 (36–60), V5 (7183) and V2 (Q52) families that are largely located proximal to D and J gene segments (Figure 3—figure supplement 1D), consistent with previous findings that hybridomas derived from fetal/neonatal B cells are bias in expressing proximal V5 (7183) and V2 (Q52) family genes (Perlmutter et al., 1985). In contrast, the splenic B-1a cells from adult animal (2–6 months) show higher frequencies in expressing distal V1 (J558) family genes including V1-75, V1-64, V1-55 and V1-53 (Figure 3—figure supplement 1D).

Collectively, we conclude that the B-1a IgH repertoire integrates rearrangements from sequential waves of de novo B-1a development that mainly occur during the first few weeks of life. The IgH repertoires defined during these waves are distinguishable both by N-additions at CDR3 junctions and by V_H_ gene usage.

### Recurring V(D)J sequences increase with age in the pre-immune B-1a IgH repertoire

**Figure 5.**
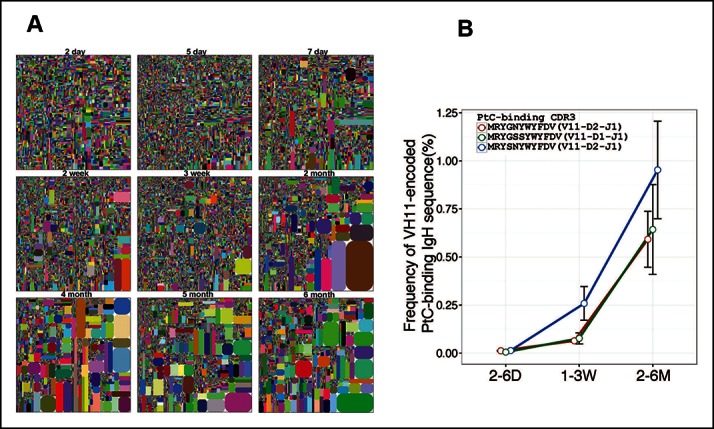
Certain V(D)J sequences increase progressively with age in the B-1a pre-immune IgH repertoire. (**A**) IgH CDR3 tree map plots for splenic B-1a samples from mice at different ages are shown. Each plot represents data for an individual mouse, except for the day 2 sample. Recurrent sequences are visualized as larger contiguously-colored rectangles in each plot. (**B**) Relative frequencies of three PtC-binding IgH CDR3 sequences in indicated splenic B-1a sample groups (n = 5–8 for each group) are plotted with mouse age. Sequence information (peptide and V(D)J recombination) is shown at the top. **DOI:** http://dx.doi.org/10.7554/eLife.09083.013

Certain V(D)J nucleotide sequences become progressively more dominant with age in the B-1a repertoire. Thus, only a lower proportion of V(D)J sequences are detected at relative higher frequency in the splenic B-1a IgH repertoire before 3 weeks, after which, both the number of recurrent sequences and the frequency at which each is represented increase progressively until the animals reach 4–6 month of age (Figure 5A, Table 2). Consequently, the distribution of the splenic B-1a IgH CDR3 nucleotide sequences diversity is much less random in adults (2–6 months) than in neonates (2–7 days) (Figure 2A).

**Figure 5—figure supplement 1.**
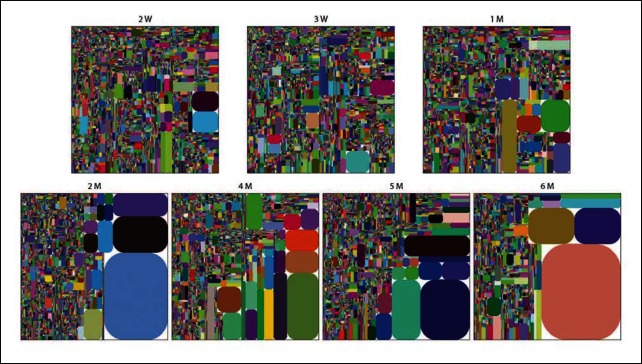
The peritoneal B-1a IgH repertoire is increasingly restricted during ontogeny. IgH CDR3 tree map plots for peritoneal B-1a samples from different ontogenic stages. Each plot represents the data for a sample from an age-defined individual mouse, except for the 2 week, 3 week and 1 month samples, which are obtained from cells pooled from several mice. Recurrent sequences are visualized as larger contiguously-colored rectangles in each plot. **DOI:** http://dx.doi.org/10.7554/eLife.09083.014

**Table 2.** Top 10 highly recurring CDR3 sequences (peptide and V(D)J recombination) detected in each of the listed splenic B-1a samples. **DOI:** http://dx.doi.org/10.7554/eLife.09083.015

sB-1a samples		Top 10 IgH CDR3 sequences		
Id	Age		Peptide	V(D)J
11168	2 weeks	1	ANDY	V1-53 J2
		2	AKHGYDAMDY	V2-9 D2-9 J4
		3	ARRYYGSSYWYFDV	V1-55 D1-1 J1
		4	ANWDY	V1-53 D4-1 J2
		5	MRYSNYWYFDV	V11-2 D2-6 J1
		6	ARDAYYWYFDV	V7-1 J1
		7	ATDYYAMDY	V1-26 J4
		8	ARFYYYGSSYAMDY	V1-55 D1-1 J4
		9	AIYYLDY	V1-53 D2-8 J2
		10	ARHYGSSYWYFDV	V2-6-2 D1-1 J1
10654	3 weeks	1	ARRYYGSSYWYFDV	V1-55 D1-1 J1
		2	ARSYSNYVMDY	V1-76 D2-6 J4
		3	ARYYGSNYFDY	V7-3 D1-1 J2
		4	ARGASYYSNWFAY	V1-55 D2-6 J3
		5	ALTGTAY	V1-53 D4-1 J3
		6	ARAGAGWYFDV	V5-9 D4-1 J1
		7	TYSNY	V6-6 D2-6 J2
		8	ARTGTYYFDY	V1-53 D4-1 J2
		9	AMVDY	V1-64 D2-9 J2
		10	ARWGTTVVGY	V1-7 D1-1 J2
7632	2 months	1	MRYGNYWYFDV	V11-2 D2-8 J1
		2	MRYSNYWYFDV	V11-2 D2-6 J1
		3	MRYGSSYWYFDV	V11-2 D1-1 J1
		4	ATFSY	V1-55 J2
		5	ARFYYYGSSYAMDY	V1-55 D1-1 J4
		6	ARIPNWVWYFDV	V1-55 D4-1 J1
		7	ARWDTTVVAPYYFDY	V1-7 D1-1 J2
		8	ARDYYGSSWYFDV	V1-26 D1-1 J1
		9	TYYDYDLYAMDY	V14-4 D2-4 J4
		10	ARFITTVVATRYWYFDV	V1-9 D1-1 J1
8699	4 months	1	ARSADYGGYFDV	V1-64 D2-4 J1
		2	ARGAY	V1-80 J2
		3	ARSYYDYPWFAY	V1-76 D2-4 J3
		4	ARRWLLNAMDY	V1-9 D2-9 J4
		5	ARPYYYGSSPWFAY	V1-69 D1-1 J3
		6	ARNDYPYWYFDV	V1-4 D2-4 J1
		7	ARSGDY	V1-64 J2
		8	ARVIGDY	V1-53 D2-14 J4
		9	ARANY	V1-55 J3
		10	AVNWDYAMDY	V1-84 D4-1 J4
8708	5 months	1	ASLTY	V1-55 J2
		2	TCNYH	V14-4 D2-8 J4
		3	LIGRNY	V1-55 D2-14 J2
		4	MRYSNYWYFDV	V11-2 D2-6 J1
		5	AKQPYYGSSYWYFDV	V2-3 D1-1 J1
		6	AGSSYAYYFDY	V1-66 D1-1 J2
		7	ARRGIDLLWYHYYAMDY	V1-26 D2-8 J4
		8	ARKSSGSRAMDY	V7-3 D3-2 J4
		9	ASYAMDY	V7-3 J4
		10	ARLYYGNSYWYFDV	V1-55 D2-8 J1
9867	6 months	1	ARKYYPSWYFDV	V1-55 D1-1 J1
		2	AREGGKFY	V1-7 J2
		3	AKSSGYAMDY	V1-55 D3-2 J4
		4	ARWVITTVARYFDV	V1-85 D1-1 J1
		5	ARGFY	V1-80 J2
		6	AKEGGYYVRAMDY	V1-55 D1-2 J4
		7	ARSMDY	V1-80 J4
		8	ASAMDY	V1-64 J4
		9	TKGGYHDYDDGAWFVY	V1-53 D2-4 J3
		10	ARKFYPSWYFDV	V1-55 J3

Table lists the top 10 highly recurring CDR3 sequences (peptide and V(D)J recombination) shown in the individual CDR3 tree-map plot of the splenic B-1a samples from 2 week to 6 month old mice (Figure 5A). For each splenic B-1a sample, the Id number and mouse age are shown in column 1 and column 2 respectively.

The recurrent V(D)J sequences include V_H_11-encoded PtC-binding V(D)J sequences, which are initially present at very low frequencies (2–6 days) but increase aggressively as animals mature to middle age (6 months) (Figure 5B). Since de novo B-1a development is minimum at adulthood, the progressive increase in the representation of the recurrent V(D)J sequences as animals reach adulthood suggests that B-1a cells are self-replenishing.

### Certain V(D)J sequences are conserved by being positively selected into the shared adult B-1a pre-immune IgH repertoire

To determine to what extent the IgH CDR3 sequences (amino acid and nucleotide) expressed by each B cell subset are shared across different individuals, we carried out CDR3 sharing analysis. In the B-1a IgH repertoire, overall, we found 30 such highly shared IgH CDR3 peptides, each of which is expressed in over 80% of the splenic B-1a samples taken from more than 20 animals with nine different ages (from 2 days to 6 months) (Table 3). Each of the shared CDR3 peptides would be expected to be encoded by several convergent V(D)J recombinations, i.e., distinct V(D)J rearrangements encode the same CDR3 amino acid sequence (Venturi et al., 2008). Strikingly, we found that each of the shared CDR3 peptides is encoded by an identical V(D)J nucleotide sequence in over 70% of splenic B-1a samples from *adult* animals (2-6 months, 9 mice) (Table 3).

**Table 3.** Certain V(D)J sequences are positively selected and conserved in adult B-1a pre-immune IgH repertoires. **DOI:** http://dx.doi.org/10.7554/eLife.09083.016

	CDR3 peptide		Predominant V(D)J		CDR3 junction diversity		Representation in indicated repertoire			
	splenic B-1a (2d-6M)		splenic B-1a (2-6M)		addition	deletion	PerC B-1a (2W-6M)	splenic B-1a (4M germ free)	FOB (2-5M)	MZB (1-5M)
1	TRWDY	17/20	V6-6 J2	8/9	TGG	J2(8)	11/11	5/6	1/8	0/7
2	MRYSNYWYFDV	17/20	V11-2 D2-6 J1	9/9	0	0	11/11	6/6	1/8	1/7
3	MRYGNYWYFDV	18/20	V11-2 D2-8 J1	9/9	0	0	11/11	6/6	1/8	1/7
4	MRYGSSYWYFDV	17/20	V11-2 D1-1 J1	9/9	0	0	11/11	6/6	1/8	1/7
5	VRHYGSSYFDY	15/20	V10-1 D1-1 J2	5/9	0	J2(1)	11/11	3/6	0/8	0/7
6	ARHYYGSSYYFDY	19/20	V5-6-1 D1-1 J2	9/9	0	0	11/11	6/6	2/8	0/7
7	ARLDY	20/20	V1-53 J2	7/9	CTg/a	J2(8)	10/11	4/6	0/8	1/7
8	ARDYYGSSYWYFDV	19/20	V7-1 D1-1 J1	6/9	0	V7-1(3)	9/11	5/6	1/8	1/7
9	ARDYYGSSWYFDV	19/20	V1-26 D1-1 J1	7/9	G	J1(3)	2/11	4/6	0/8	1/7
10	ANWDY	19/20	V14-3 D4-1 J2	6/9	0	V14-3(2)J2(8)	5/11	2/6	0/8	0/7
11	ATGTWFAY	18/20	V1-19 D4-1 J3	5/9	0	V1-19(2)	6/11	2/6	0/8	1/7
12	ARYYYGSSYAMDY	19/20	V7-3 D1-1 J4	8/9	0	V7-3(1)J4(4)	10/11	3/6	3/8	3/7
13	ARYSNYYAMDY	18/20	V1-39 D2-6 J4	6/9	0	J4(2)	8/11	1/6	0/8	0/7
14	ARDFDY	19/20	V1-64 J2	6/9	G	J2(3)	1/11	3/6	1/8	1/7
15	ARYYSNYWYFDV	17/20	V1-9 D2-6 J1	6/9	0	0	4/11	1/6	0/8	0/7
16	ARYDYDYAMDY	17/20	V1-39 D2-4 J4	6/9	0	J4(3)	7/11	1/6	0/8	0/7
17	ARHYYGSSYWYFDV	18/20	V2-6-2 D1-1 J1	6/9	0	0	6/11	2/6	1/8	3/7
18	ARFYYYGSSYAMDY	19/20	V1-55 D1-1 J4	6/9	T	J4(4)	8/11	3/6	1/8	1/7
19	ARWDFDY	19/20	V1-7 J2	6/9	TGGG	J2(3)	1/11	3/6	1/8	1/7
20	ARGAY	19/20	V1-80 J3	5/9	GGG	J3(8)	7/11	6/6	1/8	1/7
21	ARRFAY	18/20	V1-26 J3	7/9	C/A	J3(8)	9/11	3/6	1/8	1/7
22	ARRDY	18/20	V1-55 J2	5/9	AGg/a	J2(8)	6/11	3/6	1/8	1/7
23	**ASYDGYYWYFDV**	18/20	V1-55 D2-9 J1	8/9	CTATG	V1-55(1)	9/11	5/6	0/8	0/7
24	ASYAMDY	16/20	V7-3 J4	8/9	0	V7-3(5)J4(4)	9/11	6/6	0/8	1/7
25	ARRYYFDY	17/20	V1-78 J2	7/9	CGg/cT	0	8/11	2/6	0/8	0/7
26	ARNYYYFDY	15/20	V1-53 D1-2 J2	8/9	t/a	0	10/11	2/6	0/8	0/7
27	ARYYGNYWYFDV	15/20	V3-8 D2-8 J1	5/9	0	0	5/11	2/6	0/8	0/7
28	**ARRYYGSSYWYFDV**	15/20	V1-55 D1-1 J1	7/9	CGG	0	10/11	5/6	1/8	1/7
29	ARRLDY	13/20	V1-22 J2	7/9	CGAC	J2(6)	8/11	2/6	0/8	1/7
30	**ARFAY**	18/20	V1-80 J3	4/9	0	J3(4)	2/11	3/6	0/8	0/7

Column 1: CDR3 peptide sequences identified to be shared in >80% of splenic B-1a samples (20 samples from mice ranging from 2 day to 6 month old); Column 2: for each shared CDR3 peptide, a single V(D)Jrearrangement sequence is selected and conserved in over 70% of adult B-1a samples (9 samples, 2-6 month old); Columns 3 and 4: nucleotides added or deleted in CDR3 junctions; Columns 5-8: the representation of each selected V(D)J sequence within the indicate repertoires (age and number of samples are shown for each group). Rows 2-4 are PtC-binding CDR3 sequences; Row 8 is CDR3 sequence for T15 Id^+^ anti-PC antibody. The data for germ-free animals is discussed at the end of the Result section.

These V(D)J nucleotide sequences represent the IgH structures that are positively selected into the shared adult B-1a IgH repertoire among C57BL/6 mice. Although the specificities of the majority of these selected V(D)J sequences remain to be defined, they include sequences that are specific for PtC and sequence for the T15 idiotype B-1a anti-PC antibodies (Masmoudi et al., 1990). Of note, most of these V(D)J sequences have nucleotide additions and/or deletions in the CDR3 junction (Table 3), indicating that the driving force for the selection may include, but is certainly not restricted to the germline rearrangement.

The majority of the V(D)J nucleotide sequences that are conserved in the splenic B-1a IgH repertoire are also conserved in the peritoneal B-1a IgH repertoires (2W-6M, 11 samples) (Table 3). Such V(D)J nucleotide sequences, however, are rarely detectable in FOB and MZB IgH repertoires (1-5M, 7-8 samples), either because these cells do not express these CDR3 peptides or because they use different V(D)J recombination sequences to encode them (Table 3). For example, although MZB cells express antibodies encoding the same CDR3 peptide as B-1a T15-id^+^, they use different V(D)J recombinations and no single V(D)J recombination dominates within the MZB IgH repertoire (Table 4). In essence, the selection of a predominant V(D)J nucleotide sequence encoding a given CDR3 peptide is unique for the B-1a IgH repertoire.

**Table 4.** MZB IgH repertoires use different V(D)J recombination sequences to encode the same CDR3 peptide as that of B-1a anti-PC T15Id^+^. **DOI:** http://dx.doi.org/10.7554/eLife.09083.017

MZB sample Id	Age (Months)	V(D)J recombination
7630	2	V1-76 D1-1 J1 and V1-39 D1-1 J1
10658	2	V1-76 D1-1 J1
8700	4	V1-72 D1-1 J1 and V8-12 D1-1 J1
8701	5	V1-58 D1-1 J1 and V1-61 D1-1 J1
13338	4	V1-61 D1-1 J1 and V5-16 D1-1 J1

Column 1: individual MZB samples tested; column 2: age of mouse for each MZB sample; column 3: for each MZB sample, V(D)J recombination events that encode ARDYYGSSYWYFDV, which is the CDR3 peptide associated with B-1a anti-PC T15Id^+^.

### Multiple distinct V(D)J recombinations that encode the same CDR3 peptide in neonatal and young mice converge to a single identical V(D)J sequence in all adults

**Figure 6.**
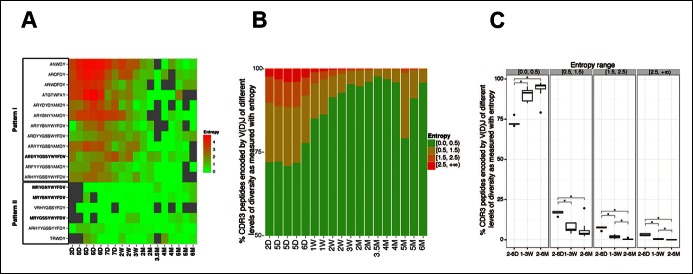
The level of convergent recombination in the B-1a IgH repertoire declines with age. (**A**) Entropy heat map showing the diversity of V(D)J recombination events for each indicated CDR3 peptide (shown at the left) in splenic B-1a samples at different ages (shown at the bottom). The higher the entropy value, the more diverse the V(D)J recombinations for a given CDR3 peptide. CDR3 peptide sequences for T15 Id^+^ anti-PC (pattern I) and anti-PtC (pattern II) antibody are shown in bold. (**B**) The diversities of the V(D)J recombination for each CDR3 peptide for the indicated splenic B-1a samples (shown at the bottom) are quantified as entropy values (see Methods and materials), which are ranked into 4 ranges (shown at the right). For each sample, the frequencies of CDR3 peptide sequences belonging to each entropy range are shown as stacks. (**C**) Splenic B-1a samples are grouped based on age. For each group (n = 5–7), the frequencies of CDR3 peptide sequences belonging to each of four entropy ranges are shown. *p<0.05, Welch’s t-test. **DOI:** http://dx.doi.org/10.7554/eLife.09083.018

In 2–7 day animals, a few selected V(D)J nucleotide sequences, such as PtC-binding sequences, have already emerged as the predominant V(D)J recombination for their corresponding CDR3 peptide (Figure 6A, pattern II). However, most of the selected V(D)J nucleotide sequences, including T15Id^+^, do not initially represent the predominant recombination for their corresponding CDR3 peptide. In particular, some CDR3 peptides are each encoded by multiple different V(D)J recombinations with similar frequencies in neonate mice. However, after weaning, a particular V(D)J recombination gradually increases its representation until it dominates in the adult B-1a IgH repertoire (Figure 6A, pattern I). In essence, although multiple distinctive V(D)J recombinations encoding the same CDR3 peptide exist in the neonatal/young B-1a IgH repertoire, a single identical V(D)J recombination sequence is selected to encode the particular CDR3 peptide in adult repertoire of almost all individuals.

**Figure 6—figure supplement 1.**
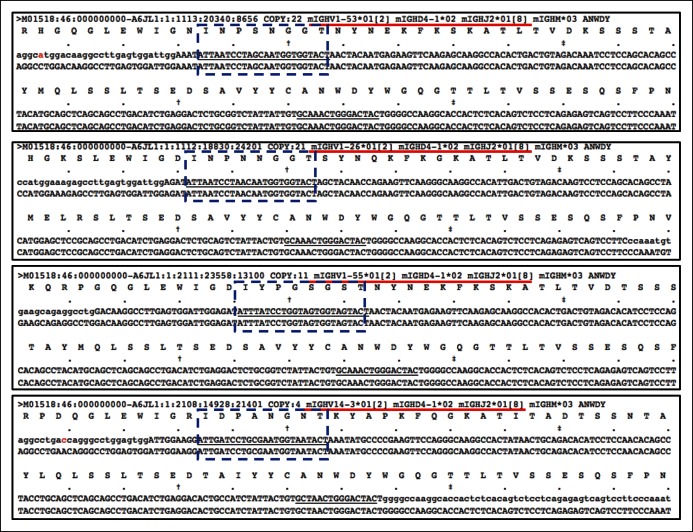
Distinct V(D)J sequences encoding the same CDR3 peptide differ in V_H_ usage. Plots showing an example, in which four different V(D)J sequences expressed by the 5 day splenic B-1a sample all encode the same CDR3 (ANWDY). Red line denotes the V(D)J recombination. CDR2 sequences are highlighted with the blue doted lined box. The V(D)J recombination (V14-3 D4-1 J2) shown in the bottom plot is the predominant V(D)J for ANWDY identified in adult splenic B-1a IgH repertoire. **DOI:** http://dx.doi.org/10.7554/eLife.09083.019

In accordance with this finding, quantification of the diversity of V(D)J recombination events for each CDR3 peptide reveals the profound convergent recombination in the neonatal B-1a IgH repertoire. Thus, about 30% of CDR3 peptide sequences in splenic B-1a IgH repertoire at 2–6 day are encoded by more than one V(D)J recombination (entropy >0.5, Figure 6B,C), and about 10% of CDR3 peptide sequences show the highest level of convergent recombination (entropy >1.5, Figure 6B,C, the higher the entropy value, the more diverse the V(D)J recombinations). However, the frequency of CDR3 peptides showing convergent recombinations steadily decrease until the animals reach adulthood (2 months), after which very few (<1%) CDR3 peptide sequences show the multiple V(D)J recombinations (entropy >1.5, Figure 6B,C).

The step-wise decreases in the level of convergent recombination as animals age indicate the potent selection that over-time shapes the B-1a IgH repertoire. In most cases, the related V(D)J sequences that ‘converge’ to encode the same CDR3 peptide share the same D and J segments but use distinct V_H_ genes (Figure 6—figure supplement 1). Therefore, despite encoding the same CDR3 peptide sequence, these related V(D)J sequences differ in their upstream regions including the CDR2 (Figure 6—figure supplement 1). These upstream differences, which can contribute to ligand binding, may be central to the selection of the predominant V(D)J sequence for the corresponding CDR3 peptide.

### AID-mediated SHM in pre-immune B-1a IgV_H_ initiates after weaning and cumulatively increases the IgH repertoire diversity thereafter

**Figure 7.**
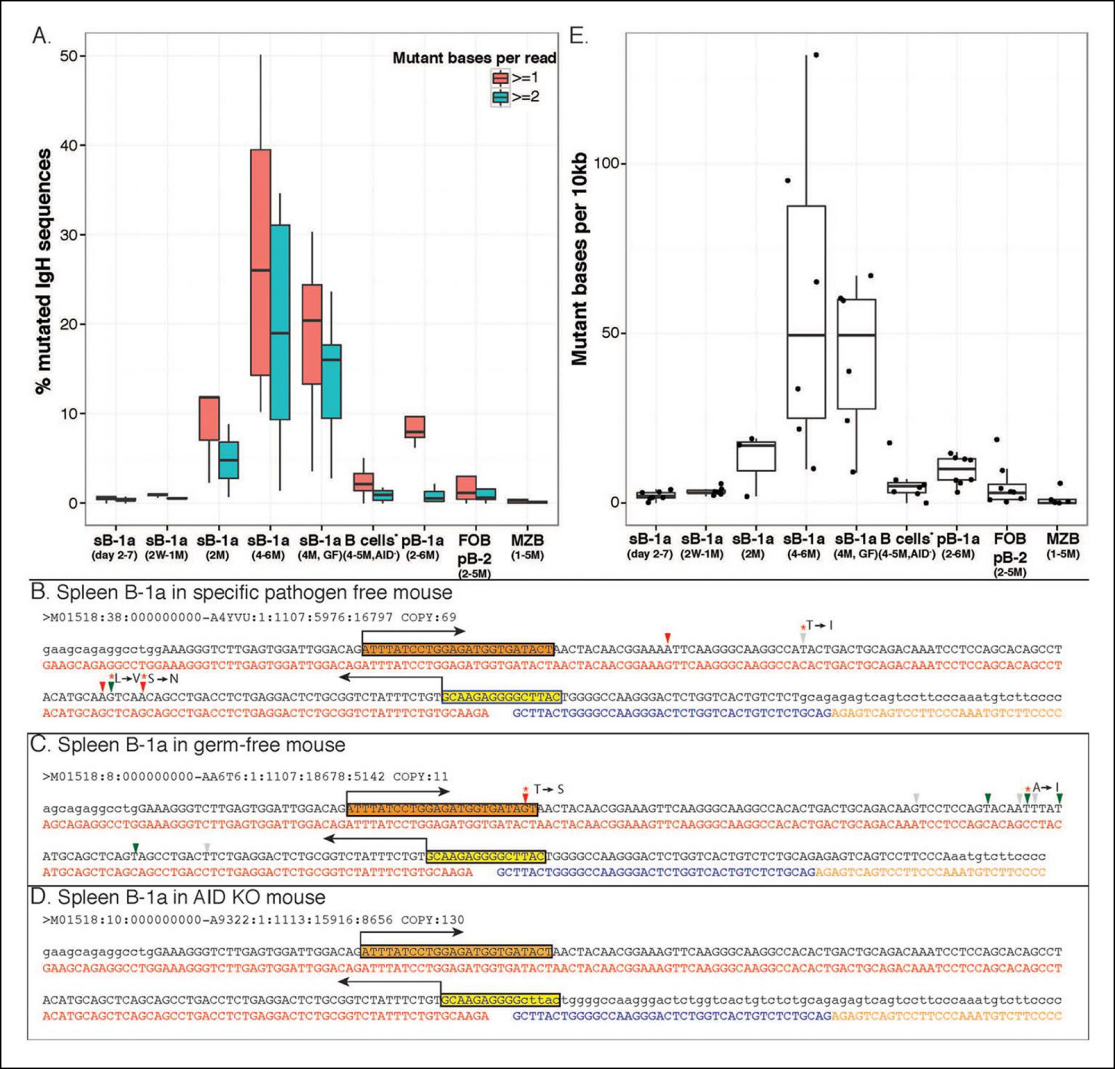
AID-mediated SHM accumulates on splenic B-1a IgV_H_ with age. (**A**) Percentages of sequences containing > = 1 (red) or > = 2 (green) nucleotide changes for B cell samples from mice at the indicated ages are shown (n = 3-8). Seven B cell samples from 4-5 month old AID knockout mice include sB-1a (n = 4), pB-1a (n = 1), FOB (n = 1) and pB-2 (n = 1). Sequences with the identical V(D)J recombination encoding ARGAY CDR3 peptide obtained from splenic B-1a sample from 4 month old specific pathogen free mouse, (**B**) germ-free mouse (**C**) and AID knockout mouse (**D**) are listed. The nucleotide substitution is analyzed at the V_H_ region stretching from the start of CDR2 (red box) to the beginning of CDR3 (yellow box). Obtained sequence (upper line) is aligned with the reference (lower line) for V1-80 (red), J3 (blue) and constant region of IgM isotype (orange). Mutations are highlighted with triangles; asterisks indicate mutations resulting in an amino acid change; red and blue triangles denote mutations in DGYW and WRCH motifs, respectively. (**E**) Numbers of mutations per 10^4^ base pairs for indicated B cell group are shown. Each dot represents data from an individual sample (n = 3–8). The data for germ-free (GF) animals is discussed at the end of the Result section. Note: The mutation profiles for the splenic B-1a IgH libraries prepared by using either old (V_H_12-3 deficient) or new primer set (V_H_12-3 included) are highly similar (Figure 7—figure supplement 3). **DOI:** http://dx.doi.org/10.7554/eLife.09083.020

Greater than 25% of splenic B-1a IgH sequences in 4–6 month old mice have at least one nucleotide change (Figure 7A). Such mutations are principally mediated by AID because they are rare (<2%) in splenic B-1a cells from age-matched AID-deficient mice (Figure 7A). The SHM even targets V(D)J sequences that are positively selected into the shared B-1a IgH repertoire in wild type mice (but not in AID-deficient mice) (Figure 7B,D). The observed mutations, most of which result in amino acid changes, are largely targeted AID hotspots, i.e., DGYW (D = A/G/T; Y = C/T; W = A/T) or WRCH (R = A/G, H = T/C/A) (Di Noia and Neuberger, 2007) (Figure 7B,C).

**Figure 7—figure supplement 1.**
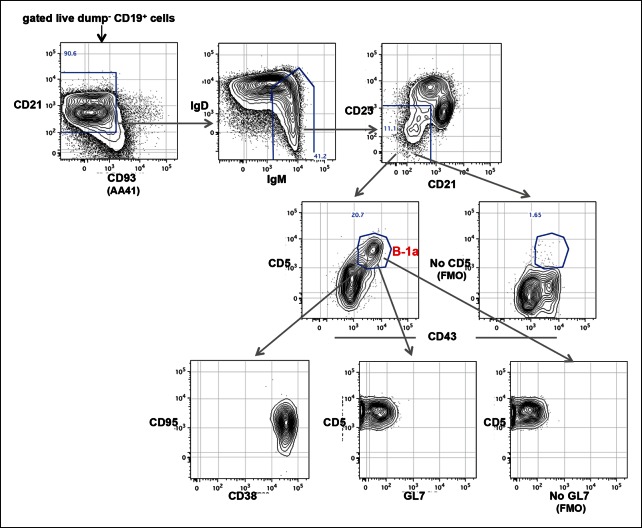
Splenic B-1a cells do not contain cells expressing GC phenotype. FACS analysis showing of live dump^-^ CD19 ^+^ CD93 (AA41)^-^ IgM^Hi^ IgD^-/lo^ CD23^-^ CD21^-/lo^ B cells from spleen of 5 month old C57BL6/J mouse were gated to reveal CD43 ^+^ CD5 ^+^ B-1a cells, which were further gated to reveal GL7, CD38 and CD95 expression. GC B cells are GL7 ^+^ CD38^-/lo^ CD95^hi^. The boundary for CD5 (*rightmost middle plot*) and GL7 (*rightmost bottom plot*) expression were determined from FMO controls in which fluorescently labeled anti-mouse CD5 or anti-mouse GL7 antibodies are omitted from the staining sets. **DOI:** http://dx.doi.org/10.7554/eLife.09083.021

**Figure 7—figure supplement 2.**
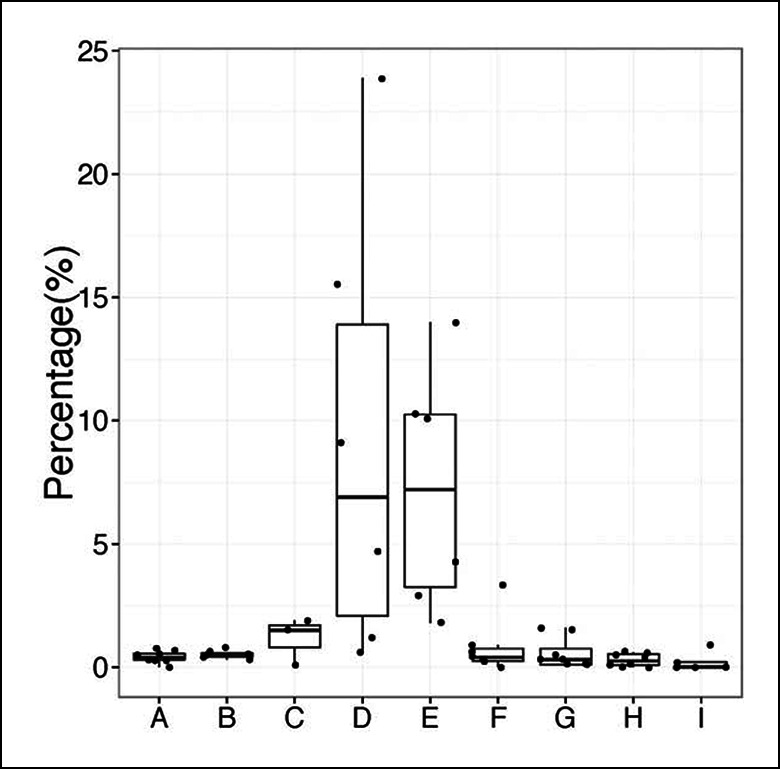
Percentage of sequences containing > = 4 nucleotides changes for each B cell group. A, sB-1a (2-7d); B, sB-1a (2W-1M); C, sB-1a (2M); D, sB-1a (4-6M); E, sB-1a (GF, 4M); F, B cells (AIDKO, 4-5M); G, pB-1a (2-6M); H, FOB, pB-2 (2-5M); I, MZB (1-5M). Each dot represents the data for an individual B cell sample, n = 3-8. **DOI:** http://dx.doi.org/10.7554/eLife.09083.022

**Figure 7—figure supplement 3.**
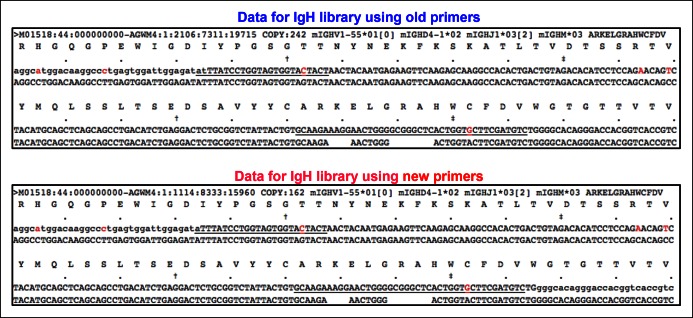
Identical V(D)J recombination sequences containing identical mutated nucleotides are detected in sequence data sets for IgH libraries obtained by using either old or new primer set. We sorted two splenic B-1a populations individually from two 4 month old C57BL/6J mice. We extracted RNA from each population and divided each RNA sample into two parts. For one part, we prepared an amplified library using the old primer set; and for the other, we prepared an amplified library using the new primer set. We then sequenced two pair of amplified IgH libraries. In two separate comparisons, we detected identical IgH sequences containing identical nucleotides substitutions in each library. One example is shown from comparing one pair of sequence data sets. Red nucleotides are the mutated bases. Upper line of sequence is the obtained sequence reads and the lower line of sequences is the V, D and J reference sequences. **DOI:** http://dx.doi.org/10.7554/eLife.09083.023

In contrast, mutations are minimal in IgV_H_ of splenic FOB, MZB and peritoneal B-2 cells from adult mice (Figure 7A). Interestingly, the frequency of mutated IgH sequences in peritoneal B-1a cells in 4-6 month old mice is substantially lower than that in age-matched splenic B-1a cells and mutations are mainly single nucleotide change (Figure 7A).

SHM in splenic B-1a IgV_H_ initiates after weaning and the frequency of mutated IgH transcripts increases with age. Thus, mutations are minimally detectable in the IgV_H_ of splenic B-1a cells from neonates (2–7 days) and young mice (2–3 weeks), are at lower frequencies in 2 month old mice, and are at substantially higher frequencies in 4–6 month old animals (Figure 7A). This age-dependent increase in splenic B-1a IgV_H_ mutation argues that the detected SHM is not due to contamination with co-sorted B cells of other subsets, including GC cells, i.e., cells with the germinal center phenotype (GL7^+^ CD38^lo^ CD95^hi^) are not detectable in the splenic B-1a population (Figure 7—figure supplement 1).

Furthermore, SHM is cumulative, becoming more pronounced with age. Thus, roughly 25% of IgH sequences from 4–6 month old splenic B-1a samples contain > = 1 nucleotide change, 19% contain > = 2 changes, and 9% contain > = 4 changes (Figure 7A and Figure 7—figure supplement 2). This translates to an average SHM rate of roughly 5 per 10^3^ base pairs (bp) (Figure 7E), the similar range as that for SHM in GC responses, i.e., 10^-3^ bp per generation (Wagner and Neuberger, 1996). Both the frequency of mutated sequences and the mutation rate for splenic B-1a samples from 2 month old mice are substantially lower than those in 4–6 month old mice (Figure 7A,E), further supporting that the SHM in the splenic B-1a IgV_H_ is an accumulative process.

### Age-dependent progressive increase in the splenic B-1a IgV_H_ mutations is accompanied by increased class-switching

Class switch recombination (CSR) is another genetic alteration process that somatically diversifies rearranged IgH genes. Both SHM and CSR are triggered by AID, which targets and introduces lesions in the IgV region for SHM and the switch regions for CSR (Muramatsu et al., 2000; Chaudhuri and Alt, 2004). Although both events require AID, SHM and CSR employ different enzymes and thus can occur independently (Li et al., 2004). Nevertheless, since they usually occur at the same differentiation stage and both are initiated by AID, the question arises as to whether the detected SHM in B-1a IgH is associated with CSR.

**Figure 8.**
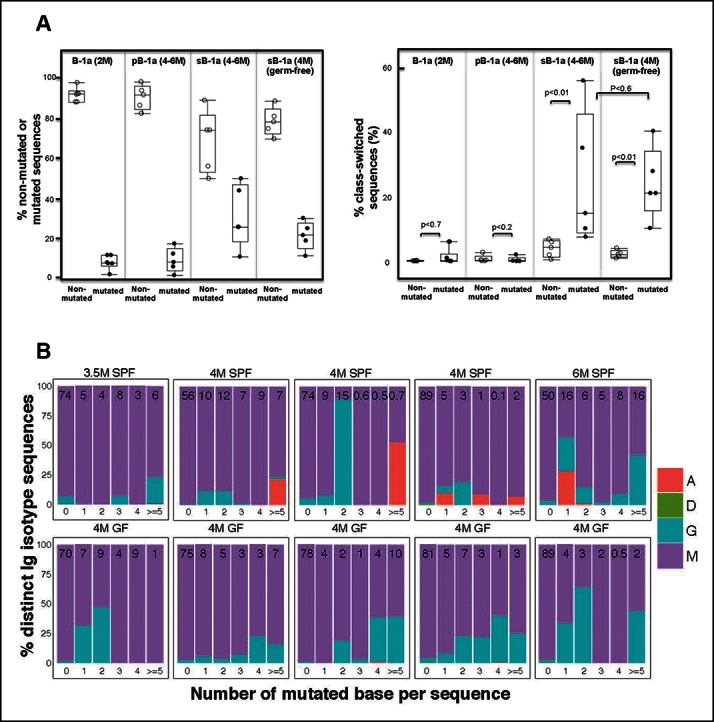
Progressive increase in the splenic B-1a IgV_H_ mutation frequency with age is accompanied by increased class-switching. (**A**) *Left panel:* The frequencies of non-mutated or mutated (> = 1 nucleotide substitution) IgH sequences obtained from indicated B cell samples are shown; *Right panel:* The frequencies of sequences expressing class-switched isotypes (neither IgM nor IgD) among non-mutated or mutated sequences are shown. Each dot represents data from an individual sample (n = 5–6). p values are calculated based on the Nonparametric Wilcoxon test. (**B**) In each plot, the IgH sequences obtained from each splenic B-1a sample from 3.5–6 month old mice are divided into five categories, based on the number of mutated nucleotides (0, 1, 2, 3, 4, > = 5) per read. In each plot, the values shown at the top are the frequencies of sequences in each category. For each category of sequences, frequencies of the distinct isotype sequences are shown as stacks. A = IgA; D = IgD; G = IgG1 + IgG3 + IgG2c + IgG2b. Each plot represents the data for a splenic B-1a sample from an individual mouse reared under either specific pathogen free (SPF) (*upper plots*) or germ-free (GF) (*lower plots*) conditions. The data for germ-free (GF) animals is discussed at the end of the Result section. **DOI:** http://dx.doi.org/10.7554/eLife.09083.024

Our method allows detection of all different Ig isotypes. For each B cell sample, we quantified the frequency of IgH sequences expressing a given isotype and examined the relationship between the isotype profiles to the mutation status. Consistent with the close relationship between CSR and SHM, wild-type B cell samples that have minimal IgV_H_ mutations, including the splenic FOB, MZB, peritoneal B-2, neonate splenic B-1a (2–7 days), young splenic and peritoneal B-1a (2–3 weeks), rarely express class-switched transcripts (Table 5). Similarly, for B cell populations that show lower levels of mutation, e.g., splenic B-1a from 2 month old animals and peritoneal B-1a from 2–6 month old animals, the majority of both mutated and non-mutated sequences are either IgM or IgD and thus rarely class-switched (Figure 8A, Table 5).

**Table 5.** B cell samples that show minimal or low level mutations in IgV_H_ rarely express class-switched transcripts. **DOI:** http://dx.doi.org/10.7554/eLife.09083.025

Sample Id	subset	age	strain	non-mutated or mutated sequences (%)		IgM (%)	IgD (%)	IgG1(%)	IgG3 (%)	IgG2c (%)	IgG2b (%)	IgE (%)	IgA (%)
13965	sB-1a	4M	AIDKO	non-mutated	98.2	99.5	0.5						
				mutated	1.8	100							
13968	sB-1a	5M	AIDKO	non-mutated	99	100							
				mutated	1	100							
13971	sB-1a	4M	AIDKO	non-mutated	98.4	99.6	0.4						
				mutated	1.6	100							
13972	sB-1a	4M	AIDKO	non-mutated	100	99.9	0.1						
8704	pB-1a	4M	AIDKO	non-mutated	98.2	99.9	0.1						
				mutated	1.8	100							
13973	pB-2	5M	AIDKO	non-mutated	100	91.6	8.4						
8700	MZB	5M	WT	non-mutated	99.8	99.9	0.1						
8701	MZB	4M	WT	non-mutated	98.6	99.9	0.1						
7630	MZB	2M	WT	non-mutated	99.5	99.9	0.1						
10658	MZB	2M	WT	non-mutated	100	100							
8702	FOB	5M	WT	non-mutated	99.8	98.6	1.4						
13966	FOB	3.5M	WT	non-mutated	99.5	99.7	0.3						
7631	FOB	2M	WT	non-mutated	99.3	72.9	27.1						
7629	pB-2	4M	WT	non-mutated	98.4	88.4	11.5						
				mutated	1.6	89	11						
13969	pB-2	3.5M	WT	non-mutated	99.5	90.1	9.9						
13974	sB-1a	day 2	WT	non-mutated	99.2	99.8	0.2						
13000	sB-1a	day 2	WT	non-mutated	99.2	100							
10659	sB-1a	day 5	WT	non-mutated	99.2	100							
9866	sB-1a	day 5	WT	non-mutated	100	100							
10651	sB-1a	day 5	WT	non-mutated	99.7	100							
10652	sB-1a	day 6	WT	non-mutated	99.4	100							
9868	sB-1a	day 7	WT	non-mutated	99.3	99.9	0.1						
9865	sB-1a	day 7	WT	non-mutated	99.5	99.6	0.4						
11168	sB-1a	2W	WT	non-mutated	99.1	99.9	0.1						
13005	sB-1a	2W	WT	non-mutated	99.5	100							
10654	sB-1a	3W	WT	non-mutated	95.8	100							
				mutated	4.2	100							
11160	pB-1a	2W	WT	non-mutated	99	100							
10655	pB-1a	3W	WT	non-mutated	99.2	100							
11163	pB-1a	1M	WT	non-mutated	99.1	99.9							
7632	sB-1a	2M	WT	non-mutated	88.1	99.1	0.9						
				mutated	11.9	99.9			0.1				
10656	sB-1a	2M	WT	non-mutated	88.1	99.8	0.2						
				mutated	11.9	100							
13004	sB-1a	2M	WT	non-mutated	97.7	99.9							
				mutated	2.3	94							6
13018	pB-1a	2M	WT	non-mutated	91.8	100							
				mutated	8	100							
13660	pB-1a	2M	WT	non-mutated	92.2	100							
				mutated	7.6	100							
7628	pB-1a	2M	WT	non-mutated	92.3	99.5	0.4		0.1				
				mutated	7.1	99.6	0.2		0.2				
8705	pB-1a	4M	WT	non-mutated	93.8	99.7	0.3						
				mutated	4.4	99.9							
9870	pB-1a	4M	WT	non-mutated	86.4	99.9							
				mutated	12.6	99.9							
11165	pB-1a	5M	WT	non-mutated	98.1	99.9							
				mutated	1.5	100							
8707	pB-1a	5M	WT	non-mutated	91.6	97.2	0.1		2	0.5	0.1		0.1
				mutated	6.2	97.9	0.1		2				
9861	pB-1a	6M	WT	non-mutated	82.4	99.6	0.4						
				mutated	17.5	100							

Table lists each individual B cell sample (labeled as distinct Id number) from wild-type (WT) or AID-deficient (AIDKO) mice. The mouse age and sample subset information are also shown. For each sample, the sequences are divided into non-mutated or mutated (> = 1 nucleotide change) categories, the frequencies of each category are shown. For each category, the frequencies of sequences with each isotype are also shown.

In contrast, both the mutated and non-mutated IgH sequences from splenic B-1a in 4–6 month old animals contain class-switched Ig (Figure 8). Importantly, the class-switched Ig (mainly IgG3, IgG2b, IgG2c and IgA) represents a significantly higher proportion of the mutated sequences than of the non-mutated sequences (Figure 8A, Table 6), indicating that the increased SHM with age in the splenic B-1a IgH repertoire is accompanied by increased class-switching. However, despite the increased class switching among mutated sequences, the frequency of class-switched sequences appears not to correlate with the increased number of mutations (Figure 8B). Consistent with the class-switching dependence on AID, we did not detect isotypes other than IgM and IgD in splenic B-1a cells from 4–5 month old AID-deficient mice (Table 5).

**Table 6.** Both the mutated and non-mutated IgH sequences obtained from splenic B-1a cells in 4-6 month old animals contain class-switched Ig. **DOI:** http://dx.doi.org/10.7554/eLife.09083.026

sample Id	subset	age	condition	non-mutated or mutated sequences (%)		IgM (%)	IgD (%)	IgG1(%)	IgG3 (%)	IgG2c (%)	IgG2b (%)	IgE(%)	IgA(%)
9867	sB-1a	6M	SPF	non-mutated	50	95.8			2.7	0.6	0.8		0.1
				mutated	50	65		0.03	16.8	4.3	4.8		9.1
8699	sB-1a	4M	SPF	non-mutated	56	99.5			0.3	0.1	0.1		
				mutated	44	89.9			3.4	2	1.3		3.4
9863	sB-1a	4M	SPF	non-mutated	74.1	93.9	0.2		3.6	1	1.3		
				mutated	25.9	44.2			41	9.7	3.7		1.4
13970	sB-1a	3.5M	SPF	non-mutated	74.1	92.7	0.5		3.7	1.7	1.3		
				mutated	25.9	92.1			2.5	0.5	4.8		0.1
13342	sB-1a	4M	SPF	non-mutated	88.9	97.6	0.5		0.8	0.6	0.2		0.3
				mutated	11.1	85.2			0.3	0.2	7.3		7
13337	sB-1a	4M	GF	non-mutated	69.7	98.5	0.1		0.8	0.4	0.1		
				mutated	30.3	79			15.3	5	0.7		
13003	sB-1a	4M	GF	non-mutated	74.8	97.2	0.3	0.2	0.5	0.2	1.6		
				mutated	25.2	89.8		1.1	2.3	1.2	5.6		
13341	sB-1a	4M	GF	non-mutated	78.2	99	0.1		0.2	0.1	0.6		
				mutated	21.8	72.2			9.6	5.5	12.6		0.1
13017	sB-1a	4M	GF	non-mutated	80.9	95.6	0.4		2	1	1		
				mutated	19.1	79	0.2		7.9	3.9	8.9		0.1
13002	sB-1a	4M	GF	non-mutated	88.5	97.4	0.5		0.6	0.2	1.3		
				mutated	11.5	63.8			14.8	8.4	13		

Table lists individual splenic B-1a cell sample sorted from 4-6 month old C57BL6/J mice reared under either specific pathogen free (SPF) or germ-free (GF) condition. For each sample, the sequences are divided into non-mutated or mutated (> = 1 nucleotide change) categories, the frequencies of each category are shown. For each category, the frequencies of sequences expressing each isotype are shown. The data for germ-free animals is discussed at the end of the result section.

The splenic B-1a cells that express class-switched Ig still express IgM on the surface, since cells were sorted as IgM^hi^ IgD^lo/-^ dump^-^ CD19^+^ CD93^-^ CD21^-/lo^ CD23^-^ CD43^+^ CD5^+^. In addition, IgM^+^ cells described here barely co-express other surface isotypes. Thus the class-switched transcripts are derived from IgM^+^ cells that apparently have already undergone class switching but have yet to turn off IgM surface protein expression. Since all of the cell preparation, staining and sorting were performed equivalently for all samples, our finding that the class-switched transcripts were selectively and predominantly detected in splenic B-1a cells from 4–6 month old mice argues that the detection of these transcripts is not due to contamination or other technical problems.

### The V(D)J selection and AID-mediated diversification that uniquely act in B-1a IgH repertoire operate comparably in germ-free and conventional mice

**Figure 9.**
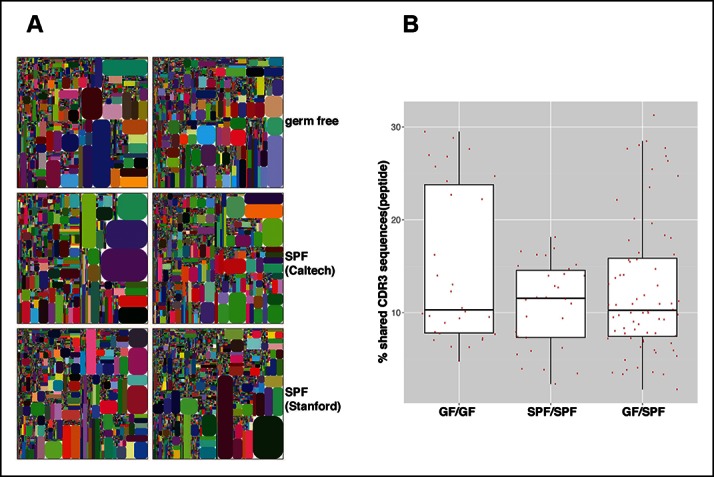
The B-1a IgH repertoires from mice raised in specific pathogen free condition are comparable to the B-1a IgH repertoire from age-matched germ-free mice. (**A**) IgH CDR3 tree map plots for splenic B-1a cells from GF mice (*upper panel*), or SPF mice in Caltech animal facility (*middle panel*), or SPF mice in Stanford animal facility (*bottom pane*l). Each plot represents the data for a sample from a 4 month old mouse. Recurrent CDR3 (nucleotide) sequences are visualized as larger contiguously-colored rectangles in each plot. (**B**) CDR3 peptide pair-wise sharing analysis of IgH repertoire similarity between multiple splenic B-1a samples from age-matched GF and SPF mice. GF mice (n = 6); SPF mice (n = 6). CDR3 peptide pair-wise analysis was conducted between GF mice (GF/GF), SPF mice (SPF/SPF) and GF vs. SPF mice (GF/SPF). Each dot represents the percentage of shared CDR3 peptide sequences between two mice. There was no statistical difference between each comparison. **DOI:** http://dx.doi.org/10.7554/eLife.09083.027

The microbiota are often thought to participate in shaping the repertoire of ‘natural’ antibodies, which is largely produced by B-1a (Baumgarth et al., 2005). Nevertheless, we find that germ-free (GF) animals have normal numbers of B-1a cells in spleen (Figure 9—figure supplement 1). Notably, the splenic B-1a IgH repertoires in age-matched (4-5 month old) specific pathogen free (SPF) and GF mice are very similar: 1) their IgH repertoires are comparably less diversified and enriched in the recurrent V(D)J sequences (Figures 2A, 9A, Table 7); 2) their V_H_ usage patterns show no significant differences (Figure 3—figure supplement 1E); 3) their CDR3 peptide expressions show a comparable extent of sharing between SPF and GF mice (Figure 9B); and 4) a substantial proportion of V(D)J sequences selected in the B-1a IgH repertoire in adult SPF mice are similarly selected in the B-1a IgH repertoire in GF mice (Table 3).

**Figure 9—figure supplement 1.**
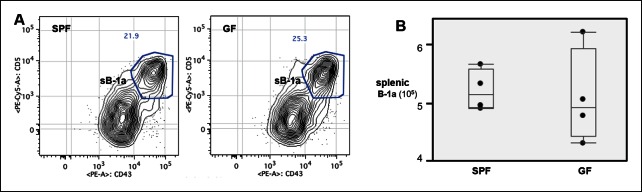
Normal splenic B-1a compartment in GF mice. (**A**) FACS plot showing the B-1a population in spleen from SPF or GF mouse. Live dump^-^ CD19 ^+^ CD93^-^ IgM^hi^ IgD^lo^ CD23^lo/-^ CD21^-^ cells were gated to reveal CD5 ^+^ CD43 ^+^ B-1a cells. (**B**) Absolute number of splenic B-1a cells in GF and SPF mice. Each dot represents the data from an individual mouse. There is no significant difference shown between two groups. **DOI:** http://dx.doi.org/10.7554/eLife.09083.028

**Table 7.** Top 10 highly recurring CDR3 sequences (peptide and V(D)J recombination) detected in listed splenic B-1a samples from age-matched SPF and GF mice. **DOI:** http://dx.doi.org/10.7554/eLife.09083.029

sB-1a samples (4 months)	Top 10 IgH CDR3 sequences		
		Peptide	V(D)J
germ-free #1	1	MRYGSSYWYFDV	V11-2 D1-1 J1
	2	ARGAY	V1-80 J2
	3	ARNPDGYYTYYYAMDY	V2-2 D2-9 J4
	4	ARDPFYYYGSSYWYFDV	V5-16 D1-1J1
	5	MRYSNYWYFDV	V11-2 D2-6 J1
	6	AITRAY	V1-55 J3
	7	ARRYYGSSYWYFDV	V1-55 D1-1 J1
	8	ARSDYYGSSSLSY	V1-26 D1-1 J2
	9	ASGGNYFDY	V1-75 J2
	10	ARSLYN	V1-9 J2
germ-free #2	1	ARNYGSSYDY	V1-53 D1-1 J2
	2	TRPSYYGSDY	V14-4 D1-1 J2
	3	TRESYDGYYVWYAMDY	V5-9-1 D2-9 J4
	4	ARGDY	V14-3 J2
	5	ASNWAY	V1-53 D4-1 J2
	6	MRYSNYWYFDV	V11-2 D2-6 J1
	7	AKGDYYGSSYYFDY	V1-9 D1-1 J2
	8	VRHGPRAFDY	V10-1 D3-2 J2
	9	ARLNGDY	V1-69 J2
	10	MRYGNYWYFDV	V11-2 D2-8 J1
specific pathogen free #1 (from Caltech)	1	ASYSNSDV	V3-6 D2-6 J1
	2	ARVSYSRAMDY	V14-3 D2-6 J4
	3	ARSGNYGAMDY	V1-7 D2-8 J4
	4	ASRLRSTFAY	V2-6-8 D1-1 J3
	5	ARVTTVHAMDY	V1-55 D1-1 J4
	6	ARNYGSSYWYFDV	V1-53 D1-1 J1
	7	ARTPNWEARDY	V1-55 D4-1 J4
	8	ARRYYGSSYWYFDV	V1-55 D1-1 J1
	9	ARPLLYRYYFDY	V1-75 D2-6 J2
	10	ARNYGSSYDWYFDV	V1-9 D1-1 J1
specific pathogen free #2 (from Caltech)	1	ARGGIYYDYDEVYYYAMDY	V1-55 D2-4 J4
	2	MRYSNYWYFDV	V11-2 D2-6 J1
	3	ARDYYGSSWYFDV	V1-26 D1-1 J1
	4	MRYGNYWYFDV	V11-2 D2-8 J1
	5	MRYGSSYWYFDV	V11-2 D1-1 J1
	6	ARYYDGYYGYYAMDY	V1-26 D2-4 J4
	7	ALITTWYFDV	V1-78 D1-2 J1
	8	ARHYYGSSWGY	V1-53 D1-1 J2
	9	ARSFSPYYFDY	V1-26 J2
	10	ARSHGYYPFDY	V1-54 D2-9 J2
specific pathogen free #1 (from Stanford)	1	ARSADYGGYFDV	V1-64 D2-4 J1
	2	ARGAY	V1-80 J2
	3	ARSYYDYPWFAY	V1-76 D2-4 J3
	4	ARRWLLNAMDY	V1-9 D2-9 J4
	5	ARPYYYGSSPWFAY	V1-69 D1-1 J3
	6	ARNDYPYWYFDV	V1-4 D2-4 J1
	7	ARSGDY	V1-64 J2
	8	ARVIGDY	V1-53 D2-14 J4
	9	ARANY	V1-55 J3
	10	AVNWDYAMDY	V1-84 D4-1 J4
specific pathogen free #2 (from Stanford)	1	ARGNY	V1-80 J2
	2	ARWVYYGSSSYWYFDV	V1-54 D1-1 J1
	3	ARSSNYAMDY	V1-78 D2-11 J4
	4	ARYYYGSNYAMDY	V7-3 D1-1 J4
	5	ARGAY	V1-80 J2
	6	ARRYYGSSYWYFDV	V1-55 D1-1 J1
	7	ARSPYYSNYEGYFDV	V1-72 D2-6 J1
	8	ARKNYGSSYWYFDV	V1-55 D1-1 J1
	9	ARLEIYYGNYGRVFDV	V1-80 D2-8 J2
	10	ARRDYYGSSYVLAY	V1-9 D1-1 J3

Table lists the top 10 highly recurring CDR3 sequences (peptide and V(D)J recombination) shown in each of CDR3 tree-map plot (Figure 9A).

Further, hypermutation occurs equally in the splenic B-1a IgV_H_ in 4–6 month old SPF and GF mice, i.e., the frequency of mutated sequences and the mutation rate are comparable under two conditions (Figure 7A,E). Indeed, AID introduces mutations into the identical V(D)J sequences expressed by splenic B-1a cells from either SPF or GF mice (Figure 7B,C). Finally, similar to SPF mice, AID-mediated class-switch occurs comparably in splenic B-1a cells from GF mice (Figure 8A). Since the V(D)J selection, hypermutation and class-switching operate comparably in splenic B-1a from GF and SPF mice, we conclude that the somatic mechanisms that select and diversify B-1a IgH repertoire over time are not driven by microbiota-derived antigens.

Nevertheless, the environment has a strong impact on the isotype representation. IgA transcripts are readily detected in splenic B-1a from 4–6 month old SPF mice; however, these transcripts are minimally detected in the splenic B-1a from age-matched GF mice (Figure 8B, Table 6). This finding is consistent with the recognition that class-switching to IgA is strongly associated with the presence of gut microbiota (Kroese et al., 1989; Macpherson et al., 2000).

### Discussion

Studies presented here open a new perspective on the origin and breadth of humoral immunity that protect against invading pathogens and regulate autoimmunity. Recent studies have already shown B-1a develops prior to and independent from BM HSC, which fail to generate B-1a but fully constitute FOB and MZB compartment (Ghosn et al., 2012; Yoshimoto et al., 2011). Cohering the fundamental difference in their development origin, our studies reveal two distinct IgH repertoires that develop at different times and are shaped by distinct functional mechanisms.

The first of these repertoires is expressed in B-1a cells. The *de novo* IgH rearrangements in this repertoire occur mainly during the first few weeks of age and largely cease thereafter. Then B-1a cells persist as a self-replenishing population. The B-1a repertoire, however, continues to evolve under stringent selection. Thus, certain V(D)J sequences increase with age, and certain V(D)J nucleotide sequences gradually emerge as the predominant recombinations encoding the specific CDR3 peptides in all adults. Furthermore, the age-dependent V(D)J selection coincides with the progressive introduction of IgV_H_ mutation and increased class-switch. Importantly, the V(D)J selection and AID-mediated diversification occur comparably in *germ-free* and conventional mice, indicating that these unique repertoire-defining mechanisms are not driven by microbiota-derived antigens.

In contrast, MZB, FOB and peritoneal B-2 cells develop later, and continuously develop *de novo* from BM HSC throughout life and express drastically different IgH repertoire(s). Their IgH repertoires tend to preferentially utilize V1 (J558) family, are far more diverse and less repetitive and, unlike B-1a cells, show no apparent selection for particular V(D)J recombination sequences and do not show IgV_H_ mutation and class-switch. In essence, AID introduces SHM and CSR in these B cell subsets only when they respond to their cognate antigens that are largely exogenous in nature.

These findings were enabled by employing the amplicon-rescued multiplex PCR technology, which allows the capture and amplification of Ig transcripts from a given B cell population in an inclusive and quantitative fashion. Specifically, the first RT-PCR reaction, which uses an array of gene-specific primers for almost all V_H_ families and all constant (C_H_) genes, is carried out only for a few cycles. The second round of PCR is then carried out with communal primers that recognize the unique sequences tagged into each of the V_H_ and C_H_ primers. Since these ‘tag sequences’ were already introduced during the initial cycles, the use of the communal primers assures that all of the targets are amplified with reduced bias during the following exponential phase of amplification. Coupled with the next generation sequencing, our method is quite robust and allows detection of diverse Ig transcripts that collectively carry about 100 V_H_ genes associated with different isotypes.

As with other bulk RNA sequence measurement, our methods cannot determine the absolute number of each Ig transcript in a given B cell population. Hence the actual number of cells expressing a certain Ig sequence is unknown. In addition, our methods do not allow determination of whether certain sequences associated with distinct isotypes belong to the same cell. Further, since the Ig transcript copy number variation among cells is unknown, the frequency of a given Ig transcript is roughly viewed as the relative index of the frequency of cells expressing this Ig transcript. This assumption is generally valid since our studies exclude plasmablast and plasma cells, which do not express surface CD5. Since B-1a cells are well-known to undergo self-replenishment in adult, the dramatic increase in certain V(D)J sequences in the B-1a IgH repertoire over time likely reflects the expansion of cells expressing this particular V(D)J sequence.

Single cell sequencing analysis has advantages in reducing technical bias and in enabling paired IgH/IgL sequencing. Nevertheless, sequencing costs are still a big hurdle to the large-scale single cell analysis, which, as our studies demonstrate, is necessary to develop a comprehensive view of the various B cell subset repertoires. Therefore, at least for the present, our approaches are more efficient and practical.

B-1a produce ‘natural’ antibodies, many of which recognize endogenous (self) antigens (Baumgarth et al., 2005) and play house-keeping roles in clearing the cellular debris or metabolic wastes (Shaw et al., 2000; Binder and Silverman, 2005). Since the natural antibodies can also react/cross-react with microorganism-derived antigens, they also participate in the first line of immune defense (Ochsenbein et al., 1999; Baumgarth et al., 2000). Germ-free mice have normal levels of circulating ‘natural’ IgM (Bos et al., 1989). Earlier immunologists have postulated that the natural antibody repertoire is selected by endogenous (self) antigens (Jerne, 1971; Coutinho et al., 1995). Our studies, which demonstrate that B-1a IgH repertoire (hence the re-activities of natural antibodies) is highly similar between individual adult C57BL/6 mice, regardless of whether the animals are reared in conventional or germ-free facilities, introduce the solid evidence supporting this argument.

Our studies also demonstrate that the B-1a IgH repertoire is selected over time. Thus, recurrent V(D)J sequences appear later, and most of the V(D)J sequences that are selected to be conserved in all individuals do not emerge until the animals reach the adulthood. As a result, the sequence composition of B-1a IgH repertoire in adult mice becomes much less random than that expressed in neonate and younger mice. Furthermore, the convergent selection of a particular V(D)J recombination sequence encoding a specific CDR3 peptide indicates that the selection is strikingly precise and occurs at both the protein and the nucleotide level.

Unexpectedly, our studies find that both SHM and CSR participate in diversifying the B-1a IgH repertoire. However, unlike GC response SHM, which occurs within a few days following antigenic stimulation, SHM in B-1a IgV_H_ starts after weaning and is cumulative with age. The progressive increase in the SHM is also associated with increased class switching. Most importantly, SHM and CRS occur comparably in *germ-free* and conventional mice, indicating that SHM and CSR in the B-1a primary IgH repertoire are not driven by microbiota-derived antigen. Since B-1a cells are well-known to produce anti-self antibodies, stimulation by endogenous antigens is likely the major driving force for the AID-mediated diversification processes.

Ongoing SHM in the absence of external antigens influence have been reported in sheep B cells (Reynaud et al., 1995). The accumulation of SHM in B-1a IgV_H_ over time likely represents a similar strategy to further diversify their restricted Ig repertoire as animal age. Such diversification may potentiate defenses against newly encountered pathogens. However, the age-dependent accumulative SHM, which is likely driven by self-antigens, may also increase the risk of autoimmune disease due to pathogenic high affinity auto-reactive antibodies. Indeed, deregulated B-1a growth have been reported in NZB/W mice, where autoantibody-associated autoimmune disease develops as animal age (Hayakawa et al., 1984).

AID-mediated mutagenesis in B-1a IgV_H_ may occasionally introduce mutations elsewhere in the genome that facilitate dysregulated growth and neoplastic transformation, e.g., B-chronic lymphocytic leukemia (B-CLL) (Stall et al., 1988; Kipps et al., 1992; Phillips and Raveche, 1992). Although the mechanism by which the IgM^+^ splenic B-1a cells from older mice express class-switched Ig transcripts remains elusive, this finding suggests that certain cells are undergoing vigorous genetic alteration that may share the similar mechanisms that underlie the malignant transformation. In fact, cells with simultaneous expression IgM and class-switched Ig transcripts have been reported in B-CLL and other B cell tumors (Oppezzo et al., 2002; Kinashi et al., 1987).

The splenic and peritoneal B-1a IgH repertoires show similar characteristics. Both repertoires become more restricted with age with increased recurrent V(D)J sequences (Figure 5—figure supplement 1) and retain the positive selected V(D)J sequences in adult animals. However, our studies reveal the key repertoire differences between B-1a cells at their two native locations. Although both repertoires show extensive CDR3 sharing among individual mice, the peritoneal B-1a IgH repertoire is more similar to neonatal splenic B-1a repertoire and shows a significantly higher level of CDR3 peptide sharing among individual mice than the splenic B-1a repertoire (Figure 2B). In addition, the peritoneal B-1a IgH repertoire is more biased in using V6-6 (J606), V9-3 (Vgam3.8), V2-9 (Q52) and V2-6-8 (Q52), which are preferentially expressed in splenic B-1a from neonate and younger mice.

These findings suggest that peritoneal B-1a cells are enriched for cells that are generated during neonatal and young age of life, thus are largely consist of cells migrated from spleen into PerC when the animals were younger. This argument is further supported by the findings that the frequencies of mutated sequences in the peritoneal B-1a cells from 4-6 month old mice are substantially lower and the mutations are mainly single nucleotide changes whereas a proportion of IgH sequences with multiple mutations is detected in splenic B-1a cells from the same aged mice (Figure 7).

MZB and B-1a share many phenotypic and functional characteristics (Martin and Kearney, 2001). Our studies show that the MZB IgH repertoire differs drastically from the B-1a IgH repertoire, but is very similar to the repertoires expressed by splenic FOB and peritoneal B-2. Since MZB and FOB cells are mainly derived from BM HSC (Ghosn et al., 2012), there findings collectively support the idea that these B cells belong to the same (i.e.,B-2) developmental lineage. Nevertheless, the MZB repertoires from individual mice contain substantially higher levels of common CDR3 sequences (peptides) than the splenic FOB and peritoneal B-2 repertoires (Figure 2B).

Years ago, we postulated that B-1a and B-2 B cells belong to distinct developmental lineages that are evolved sequentially to play complementary roles in immunity (Herzenberg and Herzenberg, 1989). The sequence data presented here, which reveal the key distinctions in the repertoires as well as the repertoire-defining mechanisms between B-1a and B-2 subsets, support this argument and greatly extend our earlier version. These key distinctions provide the genetic bases for their well-known fundamental functional difference between B-1a and other B subsets. In particular, they are central to vaccine development, where the recognition that the B cells have distinct targeting antibody repertoires clearly invites attention. In addition, our findings offer insights in understanding the origins and behaviors of B cell neoplasms, particularly B-CLL, and the autoimmune diseases in which over production of autoantibodies is implicated in the pathology.

## Materials and methods

### Mice

C57BL/6J mice were purchased from the Jackson Laboratory. AID-deficient C57BL6/J mice were kindly provided by Dr. Michel Nussenzweig (Rockefeller University). Mice were breed and kept in the Herzenberg laboratory colony under SPF conditions at the Stanford Veterinary Service Center (VSC). Spleens from germ-free C57BL6/J mice were provided by Dr. Sarkis Mazmanian (Caltech). Germ-free mice were maintained in sterile Trexler isolators and fed autoclaved food and water. Germ-free status was assayed monthly by aerobic and anaerobic plating; and by 16s rRNA PCR. Study protocols were approved by the Stanford VSC.

### Hi-dimensional FACS sorting

FACS staining has been previously described (Yang et al., 2012). Briefly, cell suspensions were incubated with LIVE/DEAD Aqua (Life Technologies, San Diego, CA), washed, and incubated with unconjugated anti-CD16/CD32 (FcRII/III) mAb to block Fc-receptors. Cells were then stained on ice for 20 min. with a ‘cocktail’ of fluorochrome-conjugated antibodies including: anti-CD21-FITC (Becton Dickenson, San Jose, CA), anti-CD43-PE (BD), anti-CD5-PE-Cy5 (BD), anti-CD19-PE-Cy5.5 (Life Technologies), anti-CD93 (AA41)-PE-Cy7 (eBioscience, San Diego, CA), anti-B220-APC (BD), anti-IgM-Alexa700 (Herzenberg lab), anti-IgD-APC-Cy7 (BioLegend, San Diego, CA), anti-CD23-Biotin (BD), anti-CD11b-PB (Life Technologies), anti-Gr-1-PB (Life Technologies), anti-TCRαβ-PB(Life Technologies), anti-CD11c-PB (Life Technologies), and anti-CD3?-PB (Life Technologies). After washing, cells were stained with Streptavidin-Qdot 605 (Life Technologies). Cells were sorted on FACS Aria (BD) at the Stanford Shared FACS Facility. Sorting purity was greater than 99%. Five types of B cell populations were sorted based on tissue and phenotype: splenic and peritoneal B-1a cells (dump^-^ CD19^+^ CD93^-^ IgM^hi^ IgD^-/lo^ CD21^-/lo^ CD23^-^ CD43^+^ CD5^+^); splenic FOB and peritoneal B-2 cells (dump^-^ CD19^+^ CD93^-^ IgM^lo^ IgD^hi^ CD23^+^ CD43^-^ CD5^-^); splenic MZB cells (dump^-^ CD19^+^ CD93^-^ IgM^hi^ IgD^-/lo^ CD21^hi^ CD23^lo/-^ CD43^-^ CD5^-^). 1-2 x 10^4^ cells for each cell population were sorted directly into 0.5 mL Trizol LS (Life Technologies).

### Amplicon rescued multiplex PCR

RNA was extracted according to the protocol provided by Trizol LS (Life Technologies). RT-PCR reactions were conducted using a set of sequence specific primers covering almost all of mouse V_H_ genes (forward primers) and constant C_H_ primers covering all of isotypes (reverse primers). Illumina paired-end sequencing communal primer B is linked to each forward V_H_ primer. Illumina paired-end sequencing communal primer A and a barcode sequence of 6 nucleotides are linked to each reverse C_H_ primers. In brief, cDNA was reverse transcribed from total RNA sample using mixture of forward V_H_ and reverse C_H_ primers and reagents from the OneStep RT-PCR kit (Qiagen, Valencia, CA). The first round of PCR was performed at: 50°C, 40 minutes; 95°C, 15 min; 94°C, 30 s, 58°C, 2 min, 72°C, 30 s, for 15 cycles; 94°C, 30 s, 72°C, 2 min, for 10 cycles; 72°C, 10 min. After the first round of PCR, primers were removed by Exonuclease I digestion at 37°C for 30 min (New England Biolabs, lpswich, MA). Then 2 μL of the first-round PCR products were used as templates for the second round of amplification using communal A and B primers and reagents from the Multiplex PCR kit (Qiagen). The second round PCR was performed as: 95°C, 15 min; 94°C, 30 s, 55°C, 30 s, 72°C, 30 s, for 40 cycles; 72°C, 5 min. About 400bp long PCR products were run on 2% agarose gels and purified using a gel extraction kit (Qiagen). The IgH libraries were pooled and sequenced with Illumina MiSeq pair-end read-length platform. The output of IgH sequence covers CDR2, CDR3 and the beginning of the constant region. The sequence information for all primers used for the library preparation can be found in US Patent Office (US9012148).

### Sequence analysis

Sequence reads were de-multiplexed according to barcode sequences at the 5’ end of reads from the IgH constant region. Reads were then trimmed according to their base qualities with a 2-base sliding window, if either quality value in this window is lower than 20, this sequence stretches from the window to 3’ end were trimmed out from the original read. Trimmed pair-end reads were joined together through overlapping alignment with a modified Needleman-Wunsch algorithm. If paired forward and reverse reads in the overlapping region were not perfectly matched, both forward and reverse reads were thrown out without further consideration. The merged reads were mapped using a Smith-Waterman algorithm to germline V, D, J and C reference sequences downloaded from the IMGT web site (Lefranc, 2003). To define the CDR3 region, the position of CDR3 boundaries of reference sequences from the IMGT database were migrated onto reads through mapping results and the resulting CDR3 regions were extracted and translated into amino acids.

### Artifacts removal

C57BL/6J mouse V_H_ reference sequences were pair-wise aligned with a Smith-Waterman algorithm. Two V_H_ reference sequences are considered related if the aligned region between them is > 200bp matched and < 6 mismatches. Two sequence reads were considered related if the best mapped V_H_ sequences are related and the CDR3 segments have less than 1 mismatch. If two sequences are related and the frequency of the minor one is less than 5% of the dominant one, the minor one is removed from further consideration. In addition, single copy CDR3s are removed from further consideration.

To allow multiplexing of multiple samples in a single sequence run, C_H_ primers were linked with barcodes containing 6 different nucleotides. The barcode C_H_ primers were used in a first round RT-PCR. To compensate for potential in chemical synthetic, PCR and/or sequencing error, barcodes were designed with a Hamming distance ≥3. Given that the chemical synthetic error is roughly 5% per position, there is about a 1/8000 chance that one barcode is mistakenly synthesized as another barcode. For a CDR3 with n occurrences in one sample and the same CDR3 (nucleotide sequence) with N occurrences in another sample in the same sequencing run, we calculated the probability that such a CDR3 would occur n or more times if it were due to cross-contamination, using the following formula $P\;=\;1\;-\;\sum_{k=1}^{n-1}\;\frac{e^{-\lambda }\;\cdot \;\lambda^{k}}{k!}$ where λ is the expected number of errors given N reads and is computed by λ = N · μ and μ is the cross-contamination rate which is preset as 1/8000. CDR3s that yielded p<0.001 were considered highly unlikely to be due to cross-contamination. Sequences were obtained for 60 separately sorted cell populations (details for each population are in Table 1).

### CDR3 tree map analysis

To draw the IgH CDR3 tree-map for each sequence sample, the entire rectangle was divided: 1^st^ into a set of rectangles with each rectangle corresponding to a distinct V_H_ gene segment; 2^nd^ into a set of V-J rectangles with each rectangle corresponding to a distinct V-J; and 3^rd^ into a set of V-J-CDR3 rectangles with each rectangle representing a distinct V-J-CDR3 combination. The rectangles are ordered based on area from largest at the bottom right to smallest at the top left. The size of an individual rectangle is proportional to the relative frequency for each V-J-CDR3 combination sequence. In order to distinguish neighboring rectangles, corners of each rectangle are rounded and each rectangles are colored randomly. Therefore, each rectangle drawn in the map represents an individual CDR3 nucleotide sequence.

### CDR3 sequence diversity (D50) measurement

D50 is a measurement of the diversity of an immune repertoire of J individuals (total number of CDR3s) composed of S distinct CDR3s in a ranked dominance configuration, where r_1_ is the abundance of the most abundant CDR3, r_2_ is the abundance of the second most abundant CDR3, and so on. C is the minimum number of distinct CDR3s with > = 50% total sequencing reads. D50 is given by

$$\begin{array}{c} Assume\;that\underset{s}{\underset{}{\;\underbrace{r_{1}\geq r_{2}\;\cdots \;\geq r_{i}\;\cdots \;\geq r_{i+1}\;\cdots \;\geq r_{s}}}}\;,\;\sum_{i=1}^{s}\;r_{i}=J\\ if\;\sum_{i=1}^{c}r_{i}\;\geq \;J/2\;and\;\sum_{i=1}^{C-1}r_{i}<\;J/2\\ D50\;=\;\frac{C}{S}\;\times \;100 \end{array}$$

### Mutation analysis

The forward V_H_ primers used to amplify expressed IgH genes are located at the IgH framework region 2. To avoid primers interfering with the mutation analysis, the variable region stretching from the beginning of the CDR2 to the beginning to the CDR3 was examined for mismatches between the sequence read and the best-aligned germline reference sequence. To eliminate the impact of sequencing error on this calculation, only sequence reads with more than 4 copies were included in the mutation calculation.

### Quantification of the diversities of V(D)J recombination events for a given CDR3 peptide

For this measurement, we introduce an entropy value as the index of diversity level. Assuming a distinct CDR3 peptide sequence X in a sample is derived from n number of distinct V(D)J recombinations (nucleotide) with each frequency as P_1_, P_2_, … P_n_ respectively, the entropy for X (*E_x_*) is then calculated as: $E_{x}\;=\;-\sum_{i=1}^{n}\;P_{i}\;\log_{2}\;P_{i}$.

For a sample, after computing entropy values for each distinct peptide CDR3 fragments, the *E* values for distinct peptide CDR3 fragments are categorized into four ranges: [0, 0.5), [0.5, 1.5), [1.5, 2.5) and [2.5, + ∞). The higher the entropy value, the more diverse the V(D)J recombinations for a given CDR3 peptide.

## Additional files

### Major datasets

The following dataset was generated:

Wang C,Yang Y,Yang Q,Herzenberg LA,Han J,2015,C57Bl/6 mice B cell subsets IgH repertoire,http://www.ncbi.nlm.nih.gov/sra/?term=SRA440320,Publicly available at NCBI Sequence Read Archive (accession no: SRA440320)

## References

[bib1] Barber CL, Montecino-Rodriguez E, Dorshkind K (2011). Reduced production of B-1-specified common lymphoid progenitors results in diminished potential of adult marrow to generate B-1 cells. Proceedings of the National Academy of Sciences.

[bib2] Baumgarth N, Herman OC, Jager GC, Brown LE, Herzenberg LA, Chen J (2000). B-1 and B-2 cell-derived immunoglobulin m antibodies are nonredundant components of the protective response to influenza virus infection. The Journal of Experimental Medicine.

[bib3] Baumgarth N, Tung JW, Herzenberg LA (2005). Inherent specificities in natural antibodies: a key to immune defense against pathogen invasion. Springer Seminars in Immunopathology.

[bib4] Baumgarth N (2011). The double life of a B-1 cell: self-reactivity selects for protective effector functions. Nature Reviews. Immunology.

[bib5] Binder CJ, Silverman GJ (2005). Natural antibodies and the autoimmunity of atherosclerosis. Springer Seminars in Immunopathology.

[bib6] Bogue M, Gilfillan S, Benoist C, Mathis D (1992). Regulation of n-region diversity in antigen receptors through thymocyte differentiation and thymus ontogeny. Proceedings of the National Academy of Sciences.

[bib7] Bos NA, Kimura H, Meeuwsen CG, de Visser H, Hazenberg MP, Wostmann BS, Pleasants JR, Benner R, Marcus DM (1989). Serum immunoglobulin levels and naturally occurring antibodies against carbohydrate antigens in germ-free BALB/c mice fed chemically defined ultrafiltered diet. European Journal of Immunology.

[bib8] Carlsson L, Holmberg D (1990). Genetic basis of the neonatal antibody repertoire: germline V-gene expression and limited N-region diversity. International Immunology.

[bib9] Chaudhuri J, Alt FW (2004). Class-switch recombination: interplay of transcription, DNA deamination and DNA repair. Nature Reviews. Immunology.

[bib10] Coutinho A, Kazatchkine MD, Avrameas S (1995). Natural autoantibodies. Current Opinion in Immunology.

[bib11] di Noia JM, Neuberger MS (2007). Molecular mechanisms of antibody somatic hypermutation. Annual Review of Biochemistry.

[bib12] Feeney AJ (1990). Lack of n regions in fetal and neonatal mouse immunoglobulin v-d-j junctional sequences. The Journal of Experimental Medicine.

[bib13] Ghosn EEB, Yamamoto R, Hamanaka S, Yang Y, Herzenberg LA, Nakauchi H, Herzenberg LA (2012). Distinct B-cell lineage commitment distinguishes adult bone marrow hematopoietic stem cells. Proceedings of the National Academy of Sciences.

[bib14] Gilfillan S, Dierich A, Lemeur M, Benoist C, Mathis D (1993). Mice lacking TdT: mature animals with an immature lymphocyte repertoire. Science.

[bib15] Gitlin AD, Shulman Z, Nussenzweig MC (2014). Clonal selection in the germinal centre by regulated proliferation and hypermutation. Nature.

[bib16] Gu H, Förster I, Rajewsky K (1990). Sequence homologies, n sequence insertion and JH gene utilization in VHDJH joining: implications for the joining mechanism and the ontogenetic timing of Ly1 b cell and b-CLL progenitor generation. The EMBO Journal.

[bib17] Hardy RR, Carmack CE, Shinton SA, Riblet RJ, Hayakawa K (1989). A single VH gene is utilized predominantly in anti-BrMRBC hybridomas derived from purified Ly-1 B cells. definition of the VH11 family. Journal of Immunology.

[bib18] Hardy RR, Hayakawa K (2001). B cell development pathways. Annual Review of Immunology.

[bib19] Hayakawa K, Hardy RR, Herzenberg LA, Herzenberg LA (1985). Progenitors for Ly-1 B cells are distinct from progenitors for other b cells. The Journal of Experimental Medicine.

[bib20] Hayakawa K, Hardy RR, Honda M, Herzenberg LA, Steinberg AD, Herzenberg LA (1984). Ly-1 b cells: functionally distinct lymphocytes that secrete IgM autoantibodies. Proceedings of the National Academy of Sciences.

[bib21] Herzenberg LA, Herzenberg LA (1989). Toward a layered immune system. Cell.

[bib22] Holodick NE, Vizconde T, Rothstein TL (2014). B-1a cell diversity: nontemplated addition in B-1a cell ig is determined by progenitor population and developmental location. Journal of Immunology.

[bib23] Jerne NK (1971). The somatic generation of immune recognition. European Journal of Immunology.

[bib24] Kantor AB, Herzenberg LA (1993). Origin of murine B cell lineages. Annual Review of Immunology.

[bib25] Kantor AB, Merrill CE, Herzenberg LA, Hillson JL (1997). An unbiased analysis of V(H)-D-J(H) sequences from B-1a, B-1b, and conventional B cells. Journal of Immunology.

[bib26] Kantor AB, Stall AM, Adams S, Herzenberg LA, Herzenberg LA (1992). Differential development of progenitor activity for three B-cell lineages. Proceedings of the National Academy of Sciences.

[bib27] Kantor AB, Stall AM, Adams S, Watanabe K, Herzenberg LA (1995). De novo development and self-replenishment of B cells. International Immunology.

[bib28] Kinashi T, Godal T, Noma Y, Ling NR, Yaoita Y, Honjo T (1987). Human neoplastic B cells express more than two isotypes of immunoglobulins without deletion of heavy-chain constant-region genes. Genes & Development.

[bib29] Kipps TJ, Rassenti LZ, Duffy S, Johnson T, Kobayashi RYO, Carson DA (1992). Immunoglobulin v gene expression in CD5 B-cell malignanciesa. Annals of the New York Academy of Sciences.

[bib30] Kirkham PM, Schroeder HW (1994). Antibody structure and the evolution of immunoglobulin V-gene segments. Seminars in Immunology.

[bib31] Kroese FG, Butcher EC, Stall AM, Lalor PA, Adams S, Herzenberg LA (1989). Many of the IgA producing plasma cells in murine gut are derived from self-replenishing precursors in the peritoneal cavity. International Immunology.

[bib32] Lalor PA, Stall AM, Adams S, Herzenberg LA (1989). Permanent alteration of the murine Ly-1 B repertoire due to selective depletion of Ly-1 B cells in neonatal animals. European Journal of Immunology.

[bib33] Lefranc M-P (2003). IMGT, the international ImMunoGeneTics database. Nucleic Acids Research.

[bib34] Li Z, Woo CJ, Iglesias-Ussel MD, Ronai D, Scharff MD (2004). The generation of antibody diversity through somatic hypermutation and class switch recombination. Genes & Development.

[bib35] Macpherson AJ, Gatto D, Sainsbury E, Harriman GR, Hengartner H, Zinkernagel RM (2000). A primitive T cell-independent mechanism of intestinal mucosal IgA responses to commensal bacteria. Science.

[bib36] Malynn BA, Yancopoulos GD, Barth JE, Bona CA, Alt FW (1990). Biased expression of JH-proximal VH genes occurs in the newly generated repertoire of neonatal and adult mice. The Journal of Experimental Medicine.

[bib37] Martin F, Kearney JF (2001). B1 cells: similarities and differences with other B cell subsets. Current Opinion in Immunology.

[bib38] Masmoudi H, Mota-Santos T, Huetz F, Coutinho A, Cazenave PA (1990). All T15 Id-positive antibodies (but not the majority of VHT15+ antibodies) are produced by peritoneal CD5+ B lymphocytes. International Immunology.

[bib39] Mercolino TJ, Arnold LW, Hawkins LA, Haughton G (1988). Normal mouse peritoneum contains a large population of Ly-1+ (cD5) B cells that recognize phosphatidyl choline. relationship to cells that secrete hemolytic antibody specific for autologous erythrocytes. The Journal of Experimental Medicine.

[bib40] Montecino-Rodriguez E, Leathers H, Dorshkind K (2006). Identification of a B-1 B cell-specified progenitor. Nature Immunology.

[bib41] Muramatsu M, Kinoshita K, Fagarasan S, Yamada S, Shinkai Y, Honjo T (2000). Class switch recombination and hypermutation require activation-induced cytidine deaminase (aID), a potential RNA editing enzyme. Cell.

[bib42] Ochsenbein AF, Fehr T, Lutz C, Suter M, Brombacher F, Hengartner H, Zinkernagel RM (1999). Control of early viral and bacterial distribution and disease by natural antibodies. Science.

[bib43] Oppezzo P, Magnac C, Bianchi S, Vuillier F, Tiscornia A, Dumas G, Payelle-Brogard B, Ajchenbaum-Cymbalista F, Dighiero G, Pritsch O (2002). Do CLL B cells correspond to naive or memory B-lymphocytes? evidence for an active Ig switch unrelated to phenotype expression and Ig mutational pattern in B-CLL cells. Leukemia.

[bib44] Perlmutter RM, Kearney JF, Chang SP, Hood LE (1985). Developmentally controlled expression of immunoglobulin VH genes. Science.

[bib45] Phillips J, Raveché E (1992). Immunoregulatory capability of murine CLL-like CD5+ B cells. Annals of the New York Academy of Sciences.

[bib46] Rajewsky K (1996). Clonal selection and learning in the antibody system. Nature.

[bib47] Reynaud CA, Garcia C, Hein WR, Weill JC (1995). Hypermutation generating the sheep immunoglobulin repertoire is an antigen-independent process. Cell.

[bib48] Seidl KJ, Mackenzie JD, Wang D, Kantor AB, Kabat EA, Herzenberg LA, Herzenberg LA (1997). Frequent occurrence of identical heavy and light chain ig rearrangements. International Immunology.

[bib49] Shaw PX, Hörkkö S, Chang MK, Curtiss LK, Palinski W, Silverman GJ, Witztum JL (2000). Natural antibodies with the T15 idiotype may act in atherosclerosis, apoptotic clearance, and protective immunity. The Journal of Clinical Investigation.

[bib50] Stall AM, Farinas MC, Tarlinton DM, Lalor PA, Herzenberg LA, Strober S, Herzenberg LA (1988). Ly-1 B-cell clones similar to human chronic lymphocytic leukemias routinely develop in older normal mice and young autoimmune (New zealand black-related) animals. Proceedings of the National Academy of Sciences.

[bib51] Tornberg UC, Holmberg D (1995). B-1a, B-1b and B-2 B cells display unique VHDJH repertoires formed at different stages of ontogeny and under different selection pressures. The EMBO Journal.

[bib52] Venturi V, Price DA, Douek DC, Davenport MP (2008). The molecular basis for public T-cell responses?. Nature Reviews. Immunology.

[bib53] Victora GD, Nussenzweig MC (2012). Germinal centers. Annual Review of Immunology.

[bib54] Wagner SD, Neuberger MS (1996). Somatic hypermutation of immunoglobulin genes. Annual Review of Immunology.

[bib55] Xu JL, Davis MM (2000). Diversity in the CDR3 region of V(H) is sufficient for most antibody specificities. Immunity.

[bib56] Yancopoulos GD, Alt FW (1986). Regulation of the assembly and expression of variable-region genes. Annual Review of Immunology.

[bib57] Yang Y, Ghosn EEB, Cole LE, Obukhanych TV, Sadate-Ngatchou P, Vogel SN, Herzenberg LA, Herzenberg LA (2012). Antigen-specific memory in B-1a and its relationship to natural immunity. Proceedings of the National Academy of Sciences.

[bib58] Yoshimoto M, Montecino-Rodriguez E, Ferkowicz MJ, Porayette P, Shelley WC, Conway SJ, Dorshkind K, Yoder MC (2011). Embryonic day 9 yolk sac and intra-embryonic hemogenic endothelium independently generate a B-1 and marginal zone progenitor lacking B-2 potential. Proceedings of the National Academy of Sciences.

